# Towards a Bio-Inspired Real-Time Neuromorphic Cerebellum

**DOI:** 10.3389/fncel.2021.622870

**Published:** 2021-05-31

**Authors:** Petruţ A. Bogdan, Beatrice Marcinnò, Claudia Casellato, Stefano Casali, Andrew G.D. Rowley, Michael Hopkins, Francesco Leporati, Egidio D'Angelo, Oliver Rhodes

**Affiliations:** ^1^Department of Computer Science, The University of Manchester, Manchester, United Kingdom; ^2^Department of Electrical, Computer and Biomedical Engineering, University of Pavia, Pavia, Italy; ^3^Neurophysiology Unit, Neurocomputational Laboratory, Department of Brain and Behavioral Sciences, University of Pavia, Pavia, Italy; ^4^IRCCS Mondino Foundation, Pavia, Italy

**Keywords:** neuromorphic computing, SpiNNaker, large scale simulation, spiking neural network, communication profiling, cerebellum model

## Abstract

This work presents the first simulation of a large-scale, bio-physically constrained cerebellum model performed on neuromorphic hardware. A model containing 97,000 neurons and 4.2 million synapses is simulated on the SpiNNaker neuromorphic system. Results are validated against a baseline simulation of the same model executed with NEST, a popular spiking neural network simulator using generic computational resources and double precision floating point arithmetic. Individual cell and network-level spiking activity is validated in terms of average spike rates, relative lead or lag of spike times, and membrane potential dynamics of individual neurons, and SpiNNaker is shown to produce results in agreement with NEST. Once validated, the model is used to investigate how to accelerate the simulation speed of the network on the SpiNNaker system, with the future goal of creating a real-time neuromorphic cerebellum. Through detailed communication profiling, peak network activity is identified as one of the main challenges for simulation speed-up. Propagation of spiking activity through the network is measured, and will inform the future development of accelerated execution strategies for cerebellum models on neuromorphic hardware. The large ratio of granule cells to other cell types in the model results in high levels of activity converging onto few cells, with those cells having relatively larger time costs associated with the processing of communication. Organizing cells on SpiNNaker in accordance with their spatial position is shown to reduce the peak communication load by 41%. It is hoped that these insights, together with alternative parallelization strategies, will pave the way for real-time execution of large-scale, bio-physically constrained cerebellum models on SpiNNaker. This in turn will enable exploration of cerebellum-inspired controllers for neurorobotic applications, and execution of extended duration simulations over timescales that would currently be prohibitive using conventional computational platforms.

## 1. Introduction

The cerebellum is an extensively studied brain area heavily involved in motor learning and coordination (Eccles et al., 1967; Ito, 2011). It can also be viewed as an area of extremes, containing both the most numerous neural cell type in the human brain—granule cell number estimated at 5 × 10^10^, ~2.5 times more numerous than the neural cells in the neocortex (Andersen et al., 2003; Shepherd, 2004; Walløe et al., 2014)—and the cell type receiving the highest number of afferent synapses—Purkinje cells can have a synaptic fan-in estimated on the order of 100,000 parallel fibers (Napper and Harvey, 1988; Tyrrell and Willshaw, 1992; Hoxha et al., 2016). This brain structure receives afferents mostly from cortex, as well as brainstem and periphery, and projects back to cortex and subcortical regions (Buckner et al., 2011; Habas and Manto, 2018).

Computer simulations of individual cell types and circuits have been designed to further understand cerebellum function. Two approaches, with two different goals, have been employed to model the cerebellum: top-down and bottom-up (Medina and Mauk, 2000; Yamazaki and Tanaka, 2005; Hausknecht et al., 2017). The top-down approach is concerned with behavior, so these models relax biological constraints. The bottom-up approach is more concerned with matching the biological nature of the underlying neurons and circuits, and has been identified as the best candidate to better understand the cerebellum (D'Angelo et al., 2016; Luque et al., 2019). For example, if one needs to implement a control system for a robotic arm for the task of sorting recyclable materials from a moving conveyor belt, then the answer currently will not involve a bottom-up analysis of cerebellar anatomy and electrophysiology of individual cells to build a system to achieve this goal. The implementation would abstract away biological details and rely on simplified mathematical models. This need not be the case. It is conceivable to merge the two views to harness the potential of biology, which satisfies both experimental results as well as higher-level requirements. One of the requirements for such a scenario is real-time simulation.

Neuromorphic computing offers the potential to simulate large-scale spiking neural network models, at speeds much faster than conventional high-performance computing (HPC) systems, and on a fraction of the power budget. However, these systems often achieve these attributes by relaxing numerical precision in mathematical operations, or by reducing the available local memory. This work explores the requirements of a biologically inspired cerebellum model, and whether it can be simulated on neuromorphic hardware. This investigation will utilize the SpiNNaker platform, as it is representative of digital neuromorphic systems in terms of constraints.

### 1.1. Contributions

High-speed and high-fidelity simulations of neural circuits hold the key to increased understanding of the circuits while simultaneously enabling engineering applications based on current knowledge of brain operation. The work presented here focuses on the cerebellum model described by Casali et al. (2019). The ability to perform real-time cerebellum simulations will benefit both the neuroscience and robotics communities. Extended duration simulations will offer insights into the long-term operation and plasticity capabilities of both the cerebellum and other neural circuits in the future, enabling simulation-based exploration of the neurophysiology of individual brain regions. Furthermore, the ability to co-simulate additional brains regions in real time, such as the cortex (Rhodes et al., 2019), will also enable research into the interaction between brain regions, e.g., the cortico-cerebellar loop. Furthermore, real-time neural simulation opens the door to exploration of brain-inspired circuits when embodied in robots, both as functional robotic controllers, and also as a path to study pathologies related to motor control.

The main contribution of this paper is the first simulation of a large-scale cerebellum model on neuromorphic hardware (SpiNNaker), yielding comparable results to a benchmark HPC-based simulator. Once validated numerically, the impact of running a data-driven cerebellum model on SpiNNaker is explored to understand the challenges of mapping and executing cerebellum circuits on neuromorphic systems. The analysis performed here will be used in the future to design a bespoke solution for real-time simulation of the cerebellum model on the SpiNNaker neuromorphic platform. Finally, a discussion on simulation capabilities and limitations of neuromorphic systems when confronted with biophysically constrained cerebellum models is presented.

## 2. Background

This section presents background on past cerebellar simulations, mainly performed on generic computing resources. The biologically representative cerebellum model used in this work is then presented in detail. Finally, the SpiNNaker neuromorphic computing platform is introduced, focusing on those characteristics which make it a suitable candidate to simulate the proposed cerebellum model.

### 2.1. Cerebellar Simulations

Several cerebellum models have been able to perform target-reaching or manipulation tasks (Carrillo et al., 2008; Luque et al., 2016; Abadia et al., 2019); replicate results of optokinetic response (Yamazaki and Nagao, 2012; Yamazaki et al., 2019), or vestibulo-ocular reflex (Naveros et al., 2018) experiments; or some combination of these (Yamazaki and Igarashi, 2013; Casellato et al., 2015; Antonietti et al., 2017, 2019). Others exposed the machine learning capabilities of a cerebellum model using six different tasks: eyelid conditioning, pendulum balancing, PID control, robot balancing, pattern recognition, and MNIST handwritten digit recognition (Hausknecht et al., 2017). These models are representative of sizes seen in the literature, ranging in scale from 10^3^ neurons and <10^6^ synapses to a billion (10^9^) neurons and synapses. The technologies required to support such a wide range of scales and model also vary in scale and specialization and can be categorized into conventional and neuromorphic computing solutions.

High-performance computing resources in the form of many core or GPU-based systems see wide application in simulating spiking neural networks thanks to their generic and flexible nature. Particularly, they can be used to simulate models of relatively complex point-neuron or very complex multi-compartment neuron models (D'Angelo et al., 2015; Florimbi et al., 2016, 2017, 2019; Torti et al., 2019). The computational capabilities required to simulate on the order of billions of neurons and synapses, such as in the case of the study by Yamazaki et al. (2019), are generally prohibitive in terms of both building and operational costs of such systems. Further, to scale up a model to such sizes requires assumptions and simplifications, which may not hold true in biological circuits. For example, the MONET simulator (Igarashi et al., 2019) used to simulate the billion neuron cerebellum model assumes communication between different cell types in the cerebellum is mostly local (in space), with little long-range interaction. Thus, the problem is transformed into one that requires sufficient parallel computing resources and avoids costly distant communication.

An alternative to generic HPC systems for spiking neural network simulation is the use of specialized, neuromorphic systems. These brain-inspired computers are designed to efficiently simulate circuits of simplified spiking neurons. Since the late 1980s, there have been a handful of neuromorphic solutions that have emerged and been assembled into large-scale systems (Furber, 2016). However, the cerebellum model used here (described by Casali et al., 2019 and presented in further detail in the following section) displays two characteristics that may be difficult or even impossible for some neuromorphic systems to tackle: large synaptic fan-ins for single cells and high peak activity arriving simultaneously at individual cells. Additionally, when explored for its motor control and learning capabilities using real or simulated robots, the cerebellum model should be executed in real time. Neuromorphic systems are optimized for accelerating the execution of spiking neural networks. In some cases, neurons and synapses were emulated in silicon on purpose-built hardware. For various reasons, including technology de-risking, cost of manufacture or identified use case, neuromorphic hardware may include a fixed and limited number of afferent synapses per neuron, thus preventing them from accurately simulating certain neural cell types in the cerebellum, such as the Purkinje cells. Individual cases are discussed below for some well-known neuromorphic systems.

The Reconfigurable On-Line Learning Spiking device (ROLLS; Qiao et al., 2015) is a real-time, full-custom, mixed-signal neuromorphic device using low power sub-threshold circuits (Chicca et al., 2014). It emulates 256 neurons and 128,000 synapses in total, that is, 512 synapses per neuron. DYNAPs (Moradi et al., 2017) sees more neurons and synapses per core, but a reduced fan-in per neuron, down to 64 synapses per neuron. In a similar range, IBM's TrueNorth neuromorphic chip (Merolla et al., 2014) of 4,096 fully digital neurosynaptic cores, each simulate 256 neurons with a fixed fan-in of 256 synapses per neuron (Cassidy et al., 2013). The HiCANN (High-Count Analogue Neural Network) chip (Schemmel et al., 2010) is an above-threshold, analogue neuromorphic implementation, which supports a fan-in of up to 14,000 synapses per neuron, although its speed-up of 10,000× compared to wall-clock time makes interfacing with robots challenging. The successor to the HiCANN chip, HiCANN2 (Schemmel et al., 2017), increases the maximum synaptic fan-in per neuron to 16,000 and enables online plasticity while reducing the speed-up to 1,000× compared to wall-clock time. The previously described systems would not be able to represent the up to 28,000 synapse fan-ins impinging onto individual Purkinje cells. Intel's Loihi chip is a real-time digital neuromorphic system flexible in terms of number of synapses supported per core up to their memory limit of 128 kB (Davies et al., 2018). Thus, by sacrificing the precision of individual weights, Loihi could represent more synapses in its synaptic memory.

### 2.2. Cerebellum Model

The cerebellum circuit selected for this work was generated from a scaffold model able to produce arbitrary volumes of mouse cerebellum (Casali et al., 2019)—the dimensions of the volume used here are 400 × 400 × 900 μm. Given the density, the hosting layer, and the soma radius, all Gloms and all cells, except for PCs, were placed within the volume using a bounded self-avoiding random walk algorithm, while PCs were placed in planar scattered arrays. The connectivity rules were based on anisotropic proximity between the pre- and post-synaptic neuronal processes and on statistical ratios of convergence and divergence. The rules used to place each neuron and produce the connectivity are described in full by Casali et al. (2019).

While the scaffold model could produce circuits of arbitrary size, this volume was selected as it had previously undergone detailed analysis in Casali et al. (2019), and is representative of the challenging spiking activity a real-time system will be required to process. Future experiments could involve larger circuits to explore scaling of the system and execution strategies.

The spiking behavior of the circuit was previously validated by Casali et al. (2019) using two popular high-precision simulators: NEST (Eppler et al., 2009) and NEURON (Hines et al., 2009). Spiking activity resulting from these simulators was compared to each other and to literature-reported values. In addition to quantitative measurements, the qualitative behavior of the model was verified to establish whether known emergent properties of the cerebellar circuit were preserved in simulation: center-surround organization and oscillation of granular layer responses, and Purkinje cell population burst-pause activity.

Given the breadth of spatiotemporal firing patterns this model replicates from experiments, an exciting next step would be to explore the model's ability to perform functional tasks: influencing and responding to environmental changes or, in other words, closing the action-perception loop. Robotics seems a natural fit for such experiments. A requirement for physical robotics experiments to take place is the hard real-time simulation of the model, meaning that all timesteps are simulated in the amount defined by the timestep (here, every 0.1 ms of neural activity should be simulated in or under 0.1 ms).

**Figure 1:**
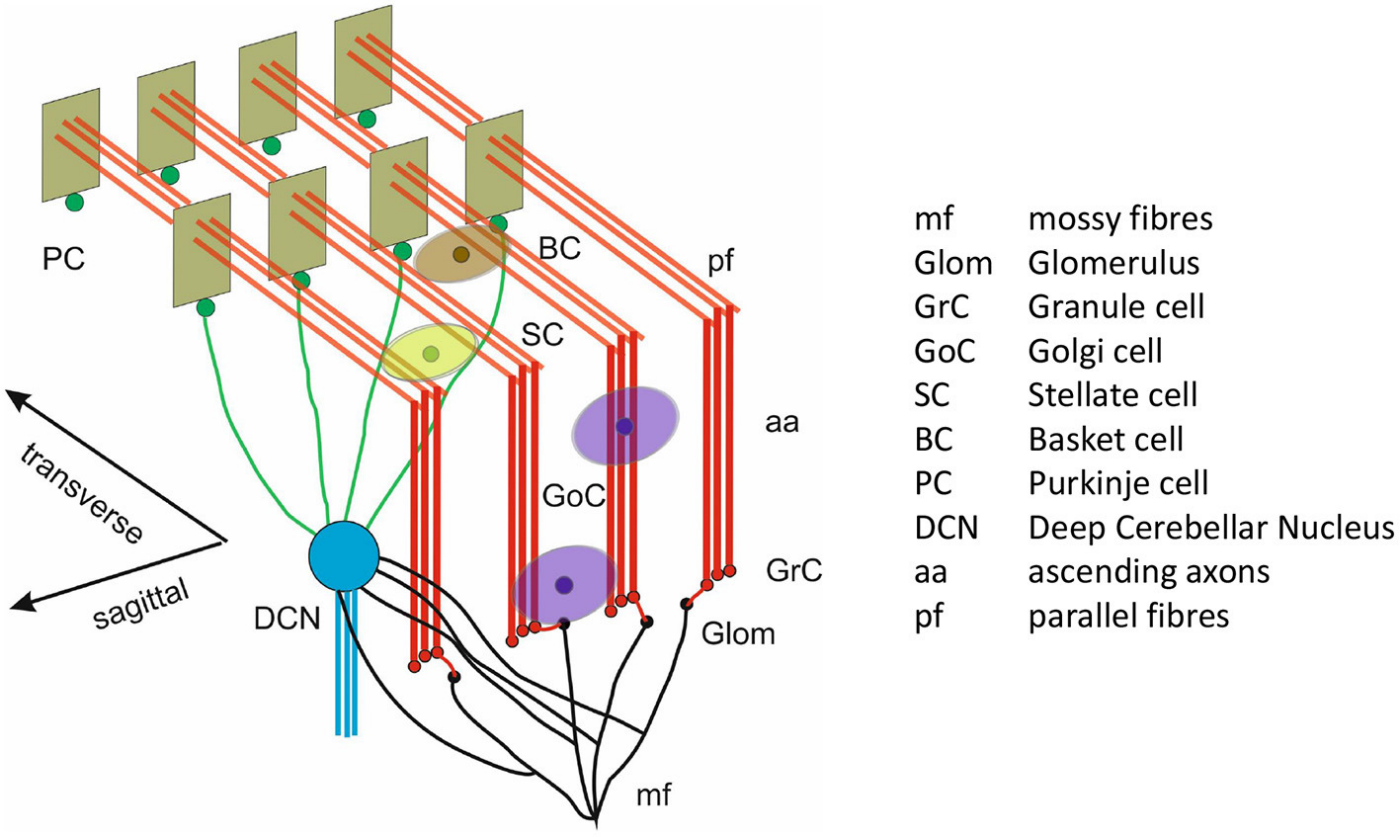
3D model architecture of the cerebellum. Reproduced from Casali et al. (2019) with permission.

The scaffold model produces the spiking network used here, as displayed in Figure 1. It is a circuit generated from a scaffold model constrained to produce 0.077mm^3^ of mouse cerebellum. The glomeruli (Glom) are the inputs of the model, while the deep cerebellar nucleus cell (DCNC) layer is the output. The population of neurons which produce excitatory projections are the granule cells (GrCs) and the populations of neurons which produce inhibitory projections are: Golgi cells (GoCs), stellate cells (SCs), basket cell (BCs), Purkinje cells (PCs), and DCNCs.

Gloms send excitatory connections to GrC (Glom-GrC), GoC (Glom-GoC), and DCNC (Glom-DCNC). Excitatory connections originating at the GrC in the form of parallel fibers (pfs) terminate at GoC (pf-GoC), PC (pf-PC), and the molecular layer interneurons SC and BC (pf-SC and pf-BC). Additional projections in the form of ascending axons (aas) connect GrC to GoC and PC. In terms of inhibitory connections these are as follows: from GoC to itself (GoC-GoC) and to GrC (GoC-GrC), PC to DCNC (PC-DCNC), and from the molecular interlayer neurons to themselves and to PC (SC-SC, BC-BC, and SC-PC, BC-PC, respectively).

The model contains a total of 96,737 neurons arranged in the seven groups or populations described previously. Table 1 summarizes the number of cells and average fan-in for each neural population in the cerebellar model. The input to the model (Glom) is composed of 7,073 non-biological neurons representing an ensemble functionally performing as a relay producing spikes when instructed to do so. Their purpose is to input activity received by the cerebellum from other brain regions. While in reality Gloms are not cells, rather large synaptic terminals, they are treated as cells for the purposes of the simulated experiments as they share attributes such as the ability to spike. The input pattern imposed onto Gloms emulated a natural sensory stimulation (Chadderton et al., 2004; Roggeri et al., 2008; Ramakrishnan et al., 2016), i.e., a 150 Hz–50 ms mossy fiber burst over a noisy background. In spatial terms, the burst was sent on a bundle of about 140 mossy fibers, each branching into about 20 Gloms.

**Table 1:** Number of cells and their average individual fan-ins (incoming synapses).

**Population**	**No. of cells**	**Average fan-in for each neuron**
Glom	7,073	N/A
GrC	88,158	6.34
GoC	219	2,060.13
SC	603	1,024.19
BC	603	1,006.47
PC	69	28,665.45
DCNC	12	173.08

The most populous group of neurons is GrC, containing 91% of all cells in the model. They also represent the main source of spikes for most other populations in the network. Of the 4.2 million synapses in the present model, 3.6 million of these originate at GrC (86% of the total synapses). The population most targeted by GrC is PC, with individual neurons there receiving, on average, 28,000 synapses from GrC. Table 2 contains the cell parameters used to configure all simulations in this work, while the connectivity parameters are presented in Table 3. Additionally, reversal potentials for conductance-based synaptic inputs are set to 0 mV for excitation and −90 mV for inhibition for all neural cell types.

**Table 2:** Cell and synapse parameters.

**Cell type**	**CM**	**IC**	***τ_m_***	***τ_refract_***	***τ_sy_n__E__***	***τ_sy_n__I__***	***v_reset_***	***v_rest_***	***v_thresh_***
GrC	0.003	0.0	2.0	1.5	0.5	10.0	−84.0	−74.0	−42.0
GoC	0.076	0.0368	21.0	2.0	0.5	10.0	−75.0	−65.0	−55.0
SC	0.0146	0.0156	14.6	1.6	0.64	2.0	−78.0	−68.0	−53.0
BC	0.0146	0.0156	14.6	1.6	0.64	2.0	−78.0	−68.0	−53.0
PC	0.62	0.6	88.0	0.8	0.5	1.6	−72.0	−62.0	−47.0
DCNC	0.089	0.0558	57.0	3.7	7.1	13.6	−69.0	−59.0	−48.0

*CM, cell membrane capacitance (nF); IC, injected current (nA). The unit for the time constants (τ_m_, τ_refract_, τ_sy_n__E__, τ_sy_n__I__) is ms. The unit for the threshold v_thresh_, rest v_rest_, and reset v_reset_ potentials is mV*.

**Table 3:** Connection parameters.

**Connection name**	**Synaptic weight (μS)**	**Delay (ms)**	**No. synapses**
Glom-GrC	9.0e-03	4	352,474
Glom-GoC	2.0e-03	4	14,302
Glom-DCNC	0.006e-03	4	1763
aa-GoC	20.0e-03	2	79,072
pf-GoC	0.4e-03	5	350,399
pf-SC	0.2e-03	5	615,177
pf-BC	0.2e-03	5	604,489
aa-PC	75.0e-03	2	17,256
pf-PC	0.02e-03	5	1,957,902
GoC-GrC	–5.0e-03	2	206,092
GoC-GoC	–8.0e-03	1	7,395
SC-SC	–2.0e-03	1	2,411
SC-PC	–8.5e-03	2	1,379
BC-BC	–2.5e-03	4	2,411
BC-PC	–9.0e-03	4	1,379
PC-DCNC	–0.03e-03	4	314

*Connections are ordered first by their afferent, pre-synaptic population, and then by their efferent, post-synaptic population. Negative weights correspond to inhibitory connections*.

### 2.3. SpiNNaker

SpiNNaker (Furber et al., 2013; Furber and Bogdan, 2020) is a digital neuromorphic computing platform, combining a multicore chip within a multicast routing fabric. The SpiNNaker chip houses 18 cores, together with Network-on-Chip (NoC) and an external RAM controller (Garside and Plana, 2020). Each core contains an ARM968 (ARM, 2004), direct memory access (DMA) controller, communications controller, network interface controller and other peripherals including a timer. Each core operates at 200 MHz clock speed, and typically runs an application simulating a group of neurons. Each core has two sets of tightly coupled memory (TCM): 32 kB for instructions (ITCM) and 64 kB for data (DTCM). Application code is compiled into an ARM968 executable and loaded to ITCM, while DTCM contains application data including heap, stack, and other read/write and zero initialized data. Each chip has an additional 128 MB of shared memory (SDRAM), directly accessible by all cores on the chip (Rhodes et al., 2018).

Individual SpiNNaker chips are assembled onto printed circuit boards in a two-dimensional, triangular mesh, with the most common board assembly consisting of 48 chips. Multiple boards can then be connected to create a SpiNNaker “machine” (Plana et al., 2020). Cores operate in a globally asynchronous locally synchronous (GALS) manner, and communicate through small messages or packets sent via the NoC and SpiNNaker router (Navaridas et al., 2015). The router allows transmission of packets to any subset of the cores on a chip, and to a subset of the six off-chip links (enabling chip-to-chip transmission, and hence routing of packets to any core on the machine). The neural applications presented herein use multicast packets, designed to be transmitted from a source to multiple targets simultaneously. A multicast packet, essentially an event encoded using the address event representation (AER, Mahowald, 1992), contains an 8-bit control byte used by the system, and a 32-bit key used to route the packet (Rhodes et al., 2018). This key is looked up within a table of entries, each of which indicates which of the matching cores and/or links packets should be sent to. This multicast behavior allows a core to send a single message targeting multiple destinations, without having to send an individual message to each of them (Rhodes et al., 2018). It also allows fire-and-forget sending of packets, removing the need for network level interlocking between source and destination. The resulting source-directed routing architecture enables highly efficient message distribution compared to traditional network architectures. A large number of simultaneous packets arriving at a router can cause it to “drop” packets, their re-sending being handled in a process called “reinjection” (Rowley et al., 2019).

Spiking neural networks are defined using the PyNN description language (Davison et al., 2008) and executed on SpiNNaker through the sPyNNaker simulator (Rhodes et al., 2018). SpiNNaker software applications are typically written in C, and compiled into ARM executable code for maximum execution speed (Rhodes et al., 2018). When designing applications which solve systems of equations, consideration must be given to the impact of precision on results and their numerical stability. The SpiNNaker ARM968 has no hardware floating-point support, and software-implemented floating-point operations are costly in terms of both ITCM and execution time. Fixed point arithmetic is therefore the preferred data representation when solving systems of equations governing neural dynamics. While creation of custom fixed point datatypes is possible, and potentially achieves optimal performance (Jin et al., 2008), the ISO/IEC TR 18037:2008 standard (ISO/IEC, 2008) is recommended and used throughout unless otherwise stated. This provides types and operators similar to those defining standard floating point operations, improving ease of reading and code development for non-specialist ARM968 programmers. Unless otherwise stated, variables in this work will be defined according to the ISO standard accum type: a signed 16-integer and 15-fractional bit fixed- point number, as discussed by Hopkins and Furber (2015).

All populations describing models have to be mapped onto SpiNNaker processing cores for simulation. The mapping process involves the partitioning, placing, and routing where populations of neurons are split into core-manageable sub-populations, which are then loaded to specific machine locations. The process through which mapping is done, as well as the effect the number of neurons placed per core have on the performance of the system are described in full by Rhodes et al. (2018).

Applications running on SpiNNaker have shown it is capable of real time simulations, inference, and learning (Bogdan et al., 2020a,b; Galluppi et al., 2020). Particularly, Rhodes et al. (2019) have simulated a large-scale, biologically representative spiking neural network in real time supported by the use of a heterogeneous parallelization scheme. The canonical cortical microcircuit model consisted of 77,000 neurons and 0.3 billion synapses and was run in real time by assigning different SpiNNaker processing cores to two distinct tasks: either processing spikes or processing neural updates. The work presented here explores whether the same organization could be employed for the cerebellum.

## 3. Materials and Methods

The model^1^ is defined using the simulator-independent description language PyNN (Davison et al., 2008) and executed on NEST (Fardet et al., 2020), and on SpiNNaker hardware using the sPyNNaker simulator (Rhodes et al., 2018).

This section contains descriptions of the experimental setup: where, how, and when stimulation is applied in the model to investigate its behavior, and the analysis methods used to validate the model behavior when simulating on SpiNNaker and NEST.

### 3.1. Experimental Setup

The model described in section 2.2 is simulated for 1 s. Initial baseline activity is driven by Gloms emitting Poisson-distributed spikes at a rate of 1 Hz. At 300 ms, stimulus is provided to a selected volume of Gloms that produce Poisson-distributed spikes at a rate of 150 Hz lasting for 50 ms. The active Gloms are selected by filtering only those cells falling within a sphere of radius 140 μm centered in the middle of the population (Casali et al., 2019). The selected volume of glomeruli corresponds to 2915 active elements, 41.21% of the total. For the final 650 ms, the input returns to the initial level with all Gloms emitting Poisson-distributed spikes at a rate of 1 Hz. These three periods of interest are defined as *pre-stimulation, stimulation* and *post-stimulation*; the same experimental conditions were used by Casali et al. (2019). This organization of the stimulus shows the firing rate of each neuron type at rest and under stimulation, and that the firing rate returns to the same level after stimulation is removed. The Poisson-based stimulus encoding method described above is a departure from the method used by Casali et al. (2019) during the stimulation period, where, during stimulation, the selected Gloms emitted synchronized spikes separated by a constant time period to achieve the prescribed firing rate. This causes large numbers of spikes to be emitted within the same timestep, which is challenging to model using SpiNNaker, and is unlikely to represent stimulation experienced by this brain region in biology (see section 4.3 for further details). Therefore, in this work, input stimulus is provided in the form of Poisson-distributed spikes matching the rates of stimulation in the original model (Casali et al., 2019), i.e., Gloms fire at 1 Hz throughout the simulation except for those selected cells firing at 150 Hz during the stimulation period.

In addition to validating the behavior of the complete cerebellum model, single cell experiments are set up to establish the modeling accuracy of each individual cell type. The first single cell experiment validates the effect of single spikes weighted by each connection type's parameters (Table 3); the delay of connections is ignored here. The second experiment validates the effect of “low” and “high” activity levels, exploring slow and fast changes in membrane potentials of each cell type; this experimental setup was employed before by Albada et al. (2018). The activity used in the latter experiment is shown to be representative of that seen in the large-scale experiment. Both single cell experiments—using single spikes and spike trains as input—model 10 s of biological time.

### 3.2. Neural Modeling

All neurons within the cerebellum model of section 2.2 are simulated via a conductance-based leaky integrate-and-fire (LIF) formulation, with sub-threshold dynamics evolving according to Equation (1).

(1) $$\begin{array}{rc} \tau_{m}\frac{dv}{dt} & =v_{rest}-v+g_{exc}\left(E_{exc}-v\right)+g_{inh}\left(E_{inh}-v\right) \end{array}$$

(2) $$\begin{array}{rc} \frac{dg}{dt} & =-\frac{g}{\tau_{syn}}+\sum g_{syn}s_{i}\left(t-d_{i}\right) \end{array}$$

Here, *v* is the membrane potential, τ_*m*_ the membrane leak time constant, *v*_*rest*_ the membrane resting potential, and *g* and *E* synaptic conductances and reversal potentials, respectively. When the membrane potential exceeds a threshold value *v*_*thr*_, a spike is emitted, and the membrane potential set to a reset potential *v*_*reset*_ for the duration of a refractory period τ_*refract*_. Synapses are either excitatory or inhibitory, both evolving over time (*t*) according to Equation (2). The second term on the right-hand side represents the incoming spikes, with $s_{i}=\underset{k}{\sum }\delta \left(t-t_{k}^{i}\right)$ representing an incoming spike train from pre-synaptic neuron *i* at time *t* subject to delay *d*_*i*_. On receiving a spike, a contribution of *g*_*syn*_ is added to the cumulative synaptic conductance *g*, before decaying with time constant τ_*syn*_.

**Figure 2:**
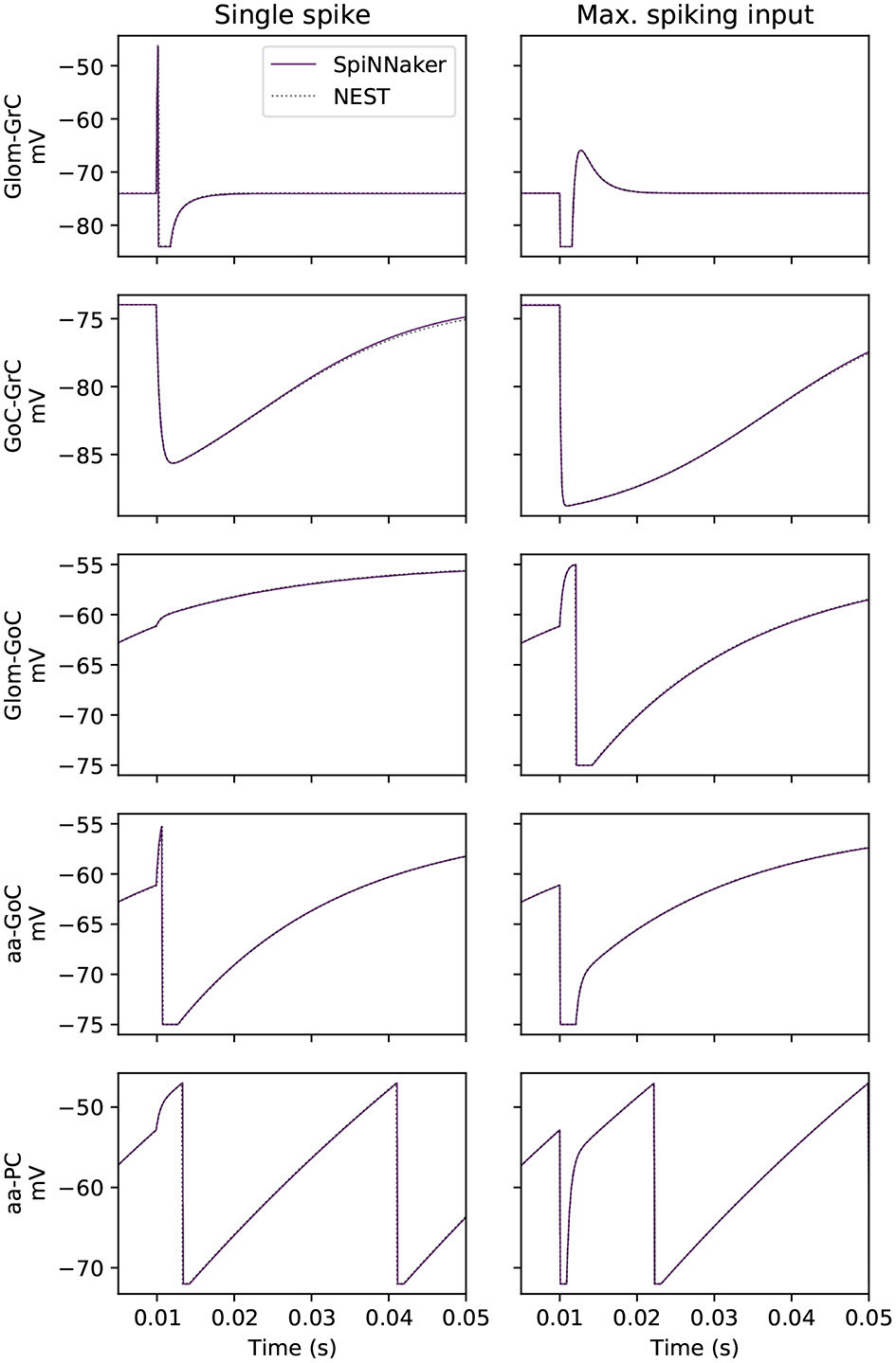
Side-by-side membrane potential traces under two experimental conditions. The experiment tests each cell type under the influence of a single spike from each possible afferent projection, a subset of which is presented here. The effect of the single spike is also scaled by the empirically recorded maximum contribution of each individual projection to single post-synaptic neurons (labeled here as “max. spiking input”).

These equations are solved in software on SpiNNaker using exact integration (Rotter and Diesmann, 1999), assuming the synaptic input remains constant over a particular timestep (Rhodes et al., 2018). This enables a sequential update of the models capturing first the synapse, and subsequently the membrane dynamics (for more details see Supplementary Material, section 1). To ensure accuracy within this solution process, a timestep of Δ*t* = 0.1 ms is used for all state updates; a larger timestep would produce less accurate results under quickly varying input conditions and it has been shown that using a 0.1 ms timestep is sufficient to capture the neural dynamics, as evidenced in Figure 2. As checks are made during this update process to determine whether neuronal membrane potential has exceeded threshold, the granularity at which spikes can be emitted in time is also resolved to the same 0.1 ms timestep.

The LIF neuron model was chosen by Casali et al. (2019) to focus on the two main construction operations of the scaffold, cell placement, and connectivity, and on the role of these latter in determining network properties while this work aimed to validate the same model with minimal differences running on neuromorphic hardware.

The cerebellum model described in section 2.2 places additional demands on the numerical solution of equations governing neuronal dynamics relative to previous studies performed on SpiNNaker. For example, while modeling has previously been validated for point neurons within cortical microcircuits (Albada et al., 2018), the conductance-based formulation together with the neuronal properties in Table 2 requires additional steps to ensure accurate modeling. The conductance-based formulation challenges the assumption that input current remains constant over a single timestep, as the synaptic input current is a function of membrane potential. Therefore, significant changes in membrane potential lead to significant changes in current, which in turn can lead to instability when updated sequentially. A sub-cycling update scheme is therefore employed to ensure numerical stability, with input current and membrane potential sequentially updated multiple times within a single timestep Δ*t*. While spikes are still emitted with Δ*t* resolution, the sub-cycling approach improves accuracy by iteratively updating neuronal state *N*_*sc*_ times within a timestep, each evolving in time by $\frac{\Delta t}{N_{sc}}$ (see Supplementary Material, section 1 for further details). This sub-cycling enables rapid changes in membrane potential to be captured accurately, while using the previously tested integration mechanisms for ODE solution on fixed-point hardware (Rhodes et al., 2018).

A challenge introduced by this sub-cycling, however, is the effective reduction in Δ*t* for each sub-cycle. When calculating membrane potential updates, care must be taken to ensure operations fit within the precision of fixed point arithmetic (section 2.3). As the simulation timestep Δ*t* is formulated into a decay factor (see Supplementary Material, section 1), reducing the effective simulation timestep through sub-cycling typically causes this decay factor to tend toward 1, which can be problematic when combined with the absolute precision available in fixed point arithmetic. Synaptic input modeling is particularly susceptible to this issue, as the relatively small quantities involved with modeling synaptic weights are susceptible to quantization during decay operations. To overcome this challenge, synaptic inputs are normalized by a conductance equal to $\frac{C_{m}}{\tau_{m}}$. This scales synaptic weights and direct current input prior to this decay process to avoid quantization, and has the added benefit of removing multiplication operations from within the neuron state update (see Supplementary Material, section 1 for further details).

The magnitudes of synaptic weights in the cerebellum network (shown in Table S1 of the Supplementary Material) cannot all be accurately represented using 16 bits when considering the need to also represent the peak synaptic input in any one timestep (also explored by Albada et al. (2018)). Section S2 of the Supplementary Material describes how the weights are represented on the machine to take into account both the minimum representable weight and the peak activity, with Table S2 revealing that all weights are representable within 5% of the prescribed weight, with the exception of pf-PC weights. These weights end up being represented by a single bit due to the relatively large peak conductance to be represented in a single timestep. Thus, pf-PC weights are rounded to the nearest representable value using a single bit, ending up at least 37% larger. Synaptic delays are typically constrained to 16 timesteps on SpiNNaker, as a buffered approach is employed for delay management (Rhodes et al., 2018). With a timestep resolution of 0.1 ms, 16 time slots provide a maximum of 1.6 ms of delay. However, the network incorporates delays of at most 5 ms, thus the limit was increased to 64 time slots, i.e., delays of at most 6.4 ms. This change is at the cost of core-local memory as previously reported by Rhodes et al. (2019).

### 3.3. Analysis Methodology

Qualitative analysis is performed for the large-scale model by presenting spike raster plots and peristimulus time histogram (PSTH) to ensure the desired model behavior is preserved when simulating on SpiNNaker. For single cell experiments, neural and synaptic traces are compared, while also reporting the lead or lag of the 100th spike. Thus, a single experiment validates both the transient behavior of cells when receiving spiking input, as well as the accuracy of the injected current integration of each cell type.

Quantitative analysis is performed based on firing rates before, during, and after stimulation. The reported firing rates are for a subset of the neurons, not an average of entire populations. This is consistent with the analysis performed by Casali et al. (2019) where values are reported for a selected subset of excited or inhibited neurons. An excited neuron is classified as one whose firing rate at least doubles during stimulation relative to pre-stimulation activity levels. Conversely, an inhibited neuron is one that exhibits a firing rate during stimulation of less than half the pre-stimulation activity level.

Furthermore, the exact limits of the stimulation intervals (pre-stimulation, stimulation, and post-stimulation) changes for each population to account for spike propagation delays from the input. Thus, the during stimulation interval start and end points are shifted by 4 ms for GrC and GoC, by 6 ms for PC, and by 9 ms for SC and BC. The DCNC is unique with the interval shifted by 10 ms to account for both excitatory and inhibitory afferent streams reaching cells in this population.

Firing rates of excited cells from each population are analyzed to extract: coefficient of variation (CV), inter-spike interval (ISI), and correlation coefficient distributions. These results are presented in the form of boxplots, highlighting the median, skewness, and outliers of each distribution to validate the behavior of the network simulated on SpiNNaker. These are computed using the electrophysiology analysis toolkit (Elephant)^2^.

Independently of neural modeling accuracy validation, this work analyzes the communication involved in the cerebellum model. The neural and synaptic update execution times have previously been documented (Rhodes et al., 2018), revealing the need to balance the number of neurons per core (here, 64 neurons per core are used for all populations, except PCs which use 1 neuron per core due to the large received peaks of activity) and number of afferents for each of these neurons. Analyzing the peak number of received multicast packets per timestep for each core is a main focus of this work as it has been shown to be the critical component when targeting real-time execution (Rhodes et al., 2019). For this, input activity is varied over multiple trials and the maximum number of received packets per core is recorded. The maximum number of packets per timestep for each core is then averaged for each population. This allows us to identify the populations, which require more resources to achieve real-time execution in the entire system. Mean number and the standard deviation for two types of stimulus are reported: Poisson-distributed spike trains and periodic, highly synchronized spikes as those originally used in simulation by Casali et al. (2019).

Finally, the mapping of cerebellum populations onto SpiNNaker is presented alongside routing information. These plots display the flow of spike packets through the network on the machine, highlighting critical paths that may become overloaded.

## 4. Results

The results presented here consist of two parts: neural spiking activity validation and analysis of communication requirements. The former involves small- and large-scale experiments simulated on SpiNNaker and NEST, with the activity produced by NEST treated as a baseline for comparison. The latter focuses on quantifying the peak number of packets produced by the validated model and the subsequent impact on individual processing cores and routers on SpiNNaker. These elements will form the basis for future optimizations to allow the hard real-time simulation of large-scale, biophysically constrained cerebellar circuits on neuromorphic systems.

### 4.1. Single Cell Validation

Before simulating the large-scale cerebellum model, behavior of individual cells in isolation is first validated. This approach offers insights into which cell types require most effort to ensure accurate dynamics, and increases confidence that the large-scale simulation will produce comparable results to the HPC baseline simulated with NEST (Eppler et al., 2009). The validation covers the two extremes experienced by the neural solvers in terms of the sub-threshold membrane potential rate of change. The membrane potential can either evolve “slowly,” when the input activity is “low,” or it can evolve “rapidly,” when the input activity is “high” (Albada et al., 2018). Cell types in the cerebellar model differ both in terms of their parameters and the number of afferents they receive, and are thus individually tested.

#### 4.1.1. Single Spike Stimulation

Validation of the effect of a single spike on all cell types in the model is performed. A source population produces a spike that arrives at the target cell at *t* = 10 ms. Target populations are created with cell parameters as defined in section 2.2 and their number is equal to the number of projections in the large-scale network. The reason for this choice is to validate the effects of single spikes weighted by each projection. The transient response to the incoming spike of the sub-threshold membrane potential of individual cells is presented in Figure 2, while the correct integration of the cell's injected current is captured by reporting the lead or lag of the 100th spike between SpiNNaker and the baseline simulation in NEST.

Figure 2 shows the effect of a single spike using the prescribed weight of the connection (left) and a scaled weight equivalent to the expected peak activity in the large-scale model (right). From the 16 total projections in the model, 5 are shown here including those targeting GrCs, as correct simulation of GrCs is observed to be crucial for that of the other neural populations as they are the most numerous and project widely throughout the model. GoCs heavily influence the activity of GrCs, so those connections which most impact (highest relative weight) GoCs are also presented (Glom-GoC and aa-GoC). Finally, the connection with the highest associated weight (aa-PC) is also included here to validate its correct behavior. The sub-threshold voltage traces match the baseline for each of the cell types presented. The tested cells produce their first spike within the same timestep as the baseline solution.

In addition to investigating the sub-threshold behavior of neurons, the relative lead or lag of the 100th spike is computed. All cells can produce spikes that are within 0.1 ms of the baseline with the exception of all tests for GrC and GoC, which do not produce 100 spikes in this experiment. In the single spike experiment, the SpiNNaker solution produces a leading spike compared to baseline for PC-DCNC by 0.1 ms, with all other cases here producing the same spike times (0 ms of lead/lag). In the maximum spiking input experiment, SpiNNaker matches baseline for all projections. Thus, SpiNNaker is shown to model accurately the neuronal response to individual spikes and injected currents.

#### 4.1.2. Representative Spike Stimulation

This test aims to validate the behavior of individual cell types using representative stimulation, with realistic input spike rates and synaptic configurations. A single cell for each type is reconstructed using the peak fan-in values from each of its afferent connections. These cells are then driven with Poisson-distributed spike trains firing at prescribed rates, as detailed in **Table 5**. The simplest example of this setup is GrC: a single GrC is created along with 2 afferent spike sources, Glom and GoC. GrCs receive up to 4 afferent synapses from Glom-GrC and GoC-GrC, thus both spike sources consist of 4 non-biological neurons each. All neurons in the Glom source fire at 144.38 Hz, while all neurons in the GoC source fire at 135.13 Hz. The most extreme example of this setup is PC, which receives 20 synapses from SC-PC, 20 from BC-PC, 278 from aa-PC, and 29,196 from pf-PC. These numbers are adjusted in all cases (not just for PC) so that any test with more than 100 source neurons is reduced to a tenth and has its firing rate increased by 10 times, i.e., pf-PC source has 2,919 GrCs firing at 895.9 Hz. Table 4 contains the full firing rates used for the two experimental scenarios: the “low” and “high” firing rate scenarios corresponding to the population rates before and during stimulation. All weights and delays used in experiments are as defined in Table 3.

**Table 4:** Single cell, realistic spikes, and connectivity test parameters and output firing rates.

**Cell type**	**Afferent**			**Input rates (Hz per neuron)**		**Low output rates (Hz)**		**High output rates (Hz)**	
	**Conn. name**	**No. of synapses**	**Conn. type**	**Low**	**High**	**SpiNNaker**	**NEST**	**SpiNNaker**	**NEST**
**GrC**	Glom–GrC	4	E	0.92	144.38	1.2	1.2	1.0	0.8
	GoC–GrC	4	I	18.63	135.13				
**GoC**	Glom–GoC	11	E	9.20	1443.80	153.4	153.1	475.7	475.5
	aa–GoC	40	E	20.50	895.90				
	pf–GoC	160	E	20.50	895.90				
	GoC–GoC	50	I	18.63	135.13				
**SC**	pf–SC	137	E	20.50	895.90	0.2	0.2	47.3	47.3
	SC–SC	11	I	31.68	220.75				
**BC**	pf–BC	134	E	20.50	895.90	0.1	0.1	27.2	27.1
	BC–BC	12	I	27.93	193.04				
**PC**	aa–PC	27	E	20.50	895.90	113.3	113.3	951.6	949.4
	pf–PC	2919	E	20.50	895.90				
	SC–PC	20	I	31.68	220.75				
	BC–PC	20	I	27.93	193.04				
**DCNC**	Glom–DCNC	14	E	0.92	144.38	15.8	15.7	0.0	0.0
	PC–DCNC	30	I	47.68	381.82				

*Cell types are tested with representative stimulation under two experimental conditions: “low” and “high” input activity. In each experiment, the input conditions are described, as well as the output firing rates produces by SpiNNaker and NEST (baseline)*.

For the low activity case, SpiNNaker produces comparable firing rates to the baseline, matching precisely for GrC, SC, BC, and PC. The other cell types produce more spikes when simulating on SpiNNaker, differing by 0.1 Hz for DCNC and by 0.3 Hz for GoC. For the high activity case, SpiNNaker matches the baseline results precisely in the case of DCNC, SC, and BC, and being within 0.2 Hz for GrC and GoC. PC firing is 1.2 Hz higher.

**Figure 3:**
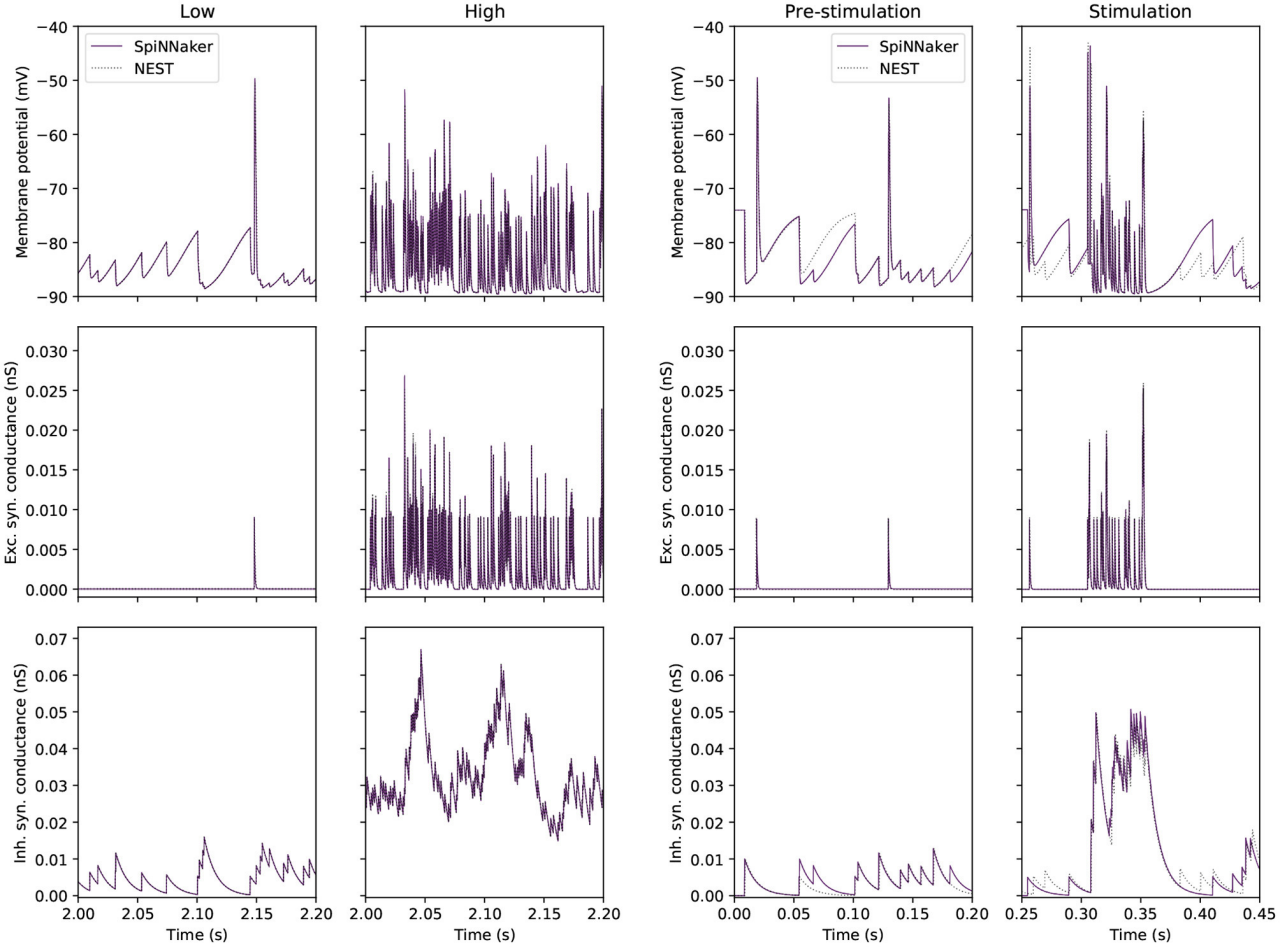
Single GrC behavior in “low” and “high” input conditions for SpiNNaker (solid) and NEST (dotted) simulations. **(Left)** Traces extracted from the single cell experiment. **(Right)** Traces extracted from a single GrC embedded in the large-scale model where the pre-stimulation period corresponds to the “low” activity case, and the stimulation period corresponds to the “high” activity case.

Figure 3 compares traces of membrane potential, and excitatory and inhibitory synaptic conductance for a tested GrC with a GrC embedded in the large-scale model. The side-by-side comparison reveals that the test conditions are representative for the range of inputs encountered in the large-scale model. On the left, the two simulations match well, as evidenced by Table 5. On the right, the mismatch between the simulations is accentuated because, in the large-scale model, small deviations from a reference point are propagated to other populations, which will amplify differences. Thus, the figure highlights the error introduced by the GrC-GoC loop influencing the selected GrC through the inhibitory conductance trace. Slight variations in the input spikes are visible in the full model, as seen in the inhibitory synaptic conductance (bottom right) panel of Figure 3. This depicts inputs to a granule cell from the Golgi population, with variations in Golgi activity introduced relative to NEST due to the combination of SpiNNaker's fixed point arithmetic, and the multiple input connections made to and within the Golgi population.

**Table 5:** Filtered firing rates for each population in a single experimental trial.

**Population**	**No. of cells**	**No. of excited (or inhibited) cells**	**Pre-stimulus (Hz) ±σ**	**Stimulus (Hz) ±σ**	**Post-stimulus (Hz) ±σ**
Glom	7,073	E_SpiNNaker_: 3,107 (43.93%)	0.92 ± 1.8	144.38 ± 62.7	0.99 ± 1.2
		E_NEST_: 3,107 (43.93%)	0.92 ± 1.8	144.38 ± 62.7	0.99 ± 1.2
GrC	88,158	E_SpiNNaker_: 16,086 (18.25%)	2.06 ± 2.7	89.59 ± 68.4	2.27 ± 2.0
		E_NEST_: 16,230 (18.41%)	2.05 ± 2.8	89.59 ± 68.2	2.21 ± 2.0
GoC	219	E_SpiNNaker_: 115 (52.51%)	18.71 ± 10.3	138.09 ± 92.1	18.83 ± 9.6
		E_NEST_: 119 (54.34%)	18.63 ± 10.1	135.13 ± 92.4	18.56 ± 9.6
SC	603	E_SpiNNaker_: 426 (70.65%)	31.94 ± 15.5	223.80 ± 82.1	31.62 ± 15.4
		E_NEST_: 424 (70.32%)	31.68 ± 15.0	220.75 ± 80.3	31.25 ± 15.3
BC	603	E_SpiNNaker_: 413 (68.49%)	28.43 ± 14.1	193.46 ± 70.7	29.48 ± 14.8
		E_NEST_: 411 (68.16%)	27.93 ± 14.0	193.04 ± 68.8	28.87 ± 14.6
PC	69	E_SpiNNaker_: 45 (65.22%)	49.60 ± 11.2	388.89 ± 149.9	51.31 ± 6.7
		E_NEST_: 44 (63.77%)	47.68 ± 9.2	381.82 ± 142.6	50.32 ± 7.3
DCNC	12	I_SpiNNaker_ : 12 (100.00%)	17.47 ± 1.6	0.00 ± 0.0	16.28 ± 1.0
		I_NEST_ : 12 (100.00%)	17.74 ± 1.6	0.00 ± 0.0	16.54 ± 0.8

*All populations except deep cerebellar nucleus cell (DCNC) report the firing rate for those neurons classified as excited for each of the simulators; DCNC only reports the firing rate for those neurons classified as inhibited*.

In summary, this experiment demonstrates that SpiNNaker produces comparable results to those produced by NEST when confronted with representative “low” and “high” activity. The firing rates produced by the simulators are within 1.2 Hz of each other in the worst-case scenario. The synaptic contribution for GrCs is accurately captured by SpiNNaker, however errors caused by the choice of arithmetic can still be seen in the computation of sub-threshold membrane potential, over time leading to divergent behavior.

### 4.2. Large-Scale Model Validation

**Figure 4:**
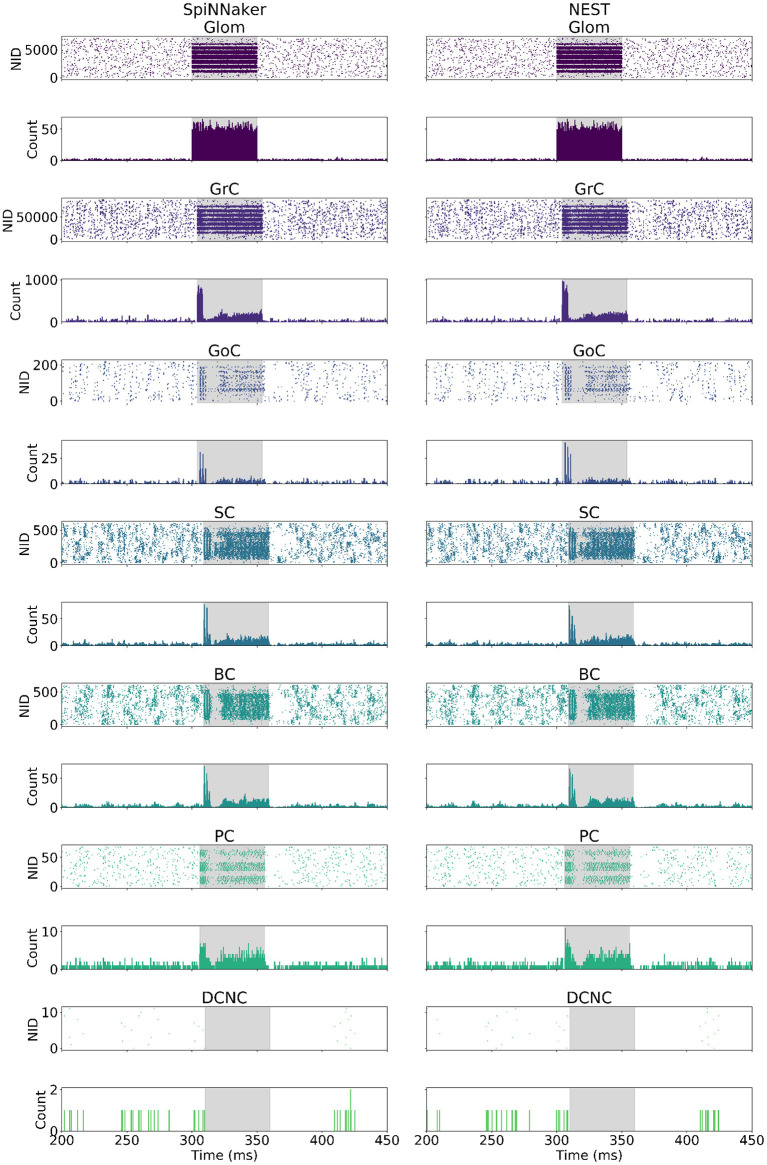
Spike raster and peristimulus time histogram (PSTH) comparison. **(Left)** SpiNNaker. **(Right)** NEST. The bin width used for the PSTH is 0.1 ms. The highlighted area corresponds to the stimulation period for each cell type. NID, neuron ID.

The cerebellum model was simulated on SpiNNaker and NEST using the same model description in PyNN, and same Poisson-distributed spiking activity as input. The spiking activity of all cells in the model is presented in Figure 4, in the form of raster plots, as well as a peristimulus time histogram binning the activity in each timestep (0.1 ms). Figure 4, provides a side-by-side comparison of the spiking network activity produced by each of the two simulators. Both simulations show the large input burst, lasting from 300 to 350 ms originating at Glom, giving rise to a peak in firing rate in all other populations with the exception of DCNC. The initial increase in firing rate is followed by a sharp decrease following GoC activation, further succeeded by a rebound in activity. DCNCs are fully silenced throughout stimulation, while post-stimulation there is a pause in the activity of GoC, SC, BC, and PC.

The neural population responses before, during, and after stimulation for the individual simulations shown in Figure 4 are summarized in Table 5. As described in section 3.3, the values reported in the table are firing rate averages of either excited or inhibited cells. Table 5, also includes the absolute number of excited (E_SpiNNaker_ and E_NEST_) or inhibited (I_SpiNNaker_ and I_NEST_) cells, as well as their relative proportion of the entire population.

- Glom had a mean firing rate of 0.92 Hz before, 0.99 Hz after stimulation, and 140 Hz during burst stimulation, closely aligned to the values reported by Casali et al. (2019). The small difference to the values reported there are because a different set of spikes are generated for the work presented here. Both the SpiNNaker and NEST simulations are driven using identical spike trains.
- GrCs spiked at an average of 2 Hz at rest and 89 Hz during stimulation. Both the SpiNNaker and NEST simulation produced over 18% of excited GrCs. The two simulations differ by 144 cells being classified as excited (0.16% of all GrCs).
- GoCs spiked at an average of 18 Hz at rest and 138 Hz during stimulation on SpiNNaker. The SpiNNaker and NEST simulations differ here by 3 Hz and 4 excited cells (1.8% of all GoCs).
- SCs spiked at an average of 32 Hz at rest and 223 Hz during stimulation. The SpiNNaker and NEST simulations differ here by 3.1 Hz and 2 excited cells (0.3% of all SCs).
- BCs spiked at an average of 28 Hz at rest and 193 Hz during stimulation. SpiNNaker sees an increase of just 0.4 Hz compared to NEST and 2 excited cells (0.3% of all BCs).
- PCs spiked at an average of 49 Hz at rest and 388 Hz during stimulation on SpiNNaker, but 381 Hz on average on NEST. This is a difference of 7 Hz with 1 more excited cell for SpiNNaker (1.5% of all PCs).
- DCNCs spiked at an average of 17 Hz at rest and 0 Hz during stimulation. Both SpiNNaker and NEST produce the same number of inhibited cells here, with 100% of DCNCs silent during stimulation.

**Figure 5:**
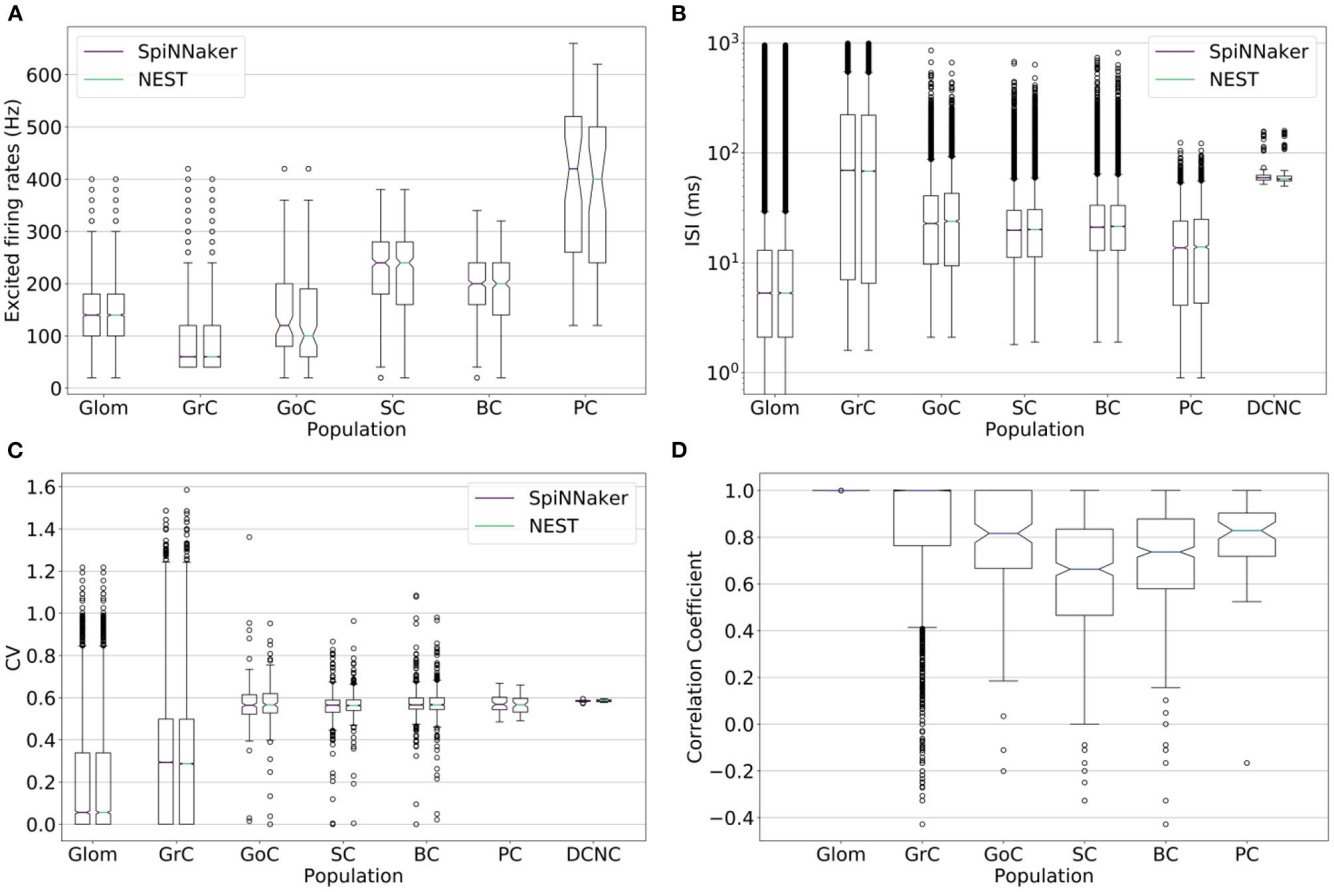
**(A)** Firing rate, **(B)** inter-spike interval (ISI), **(C)** coefficient of variation (CV), and **(D)** correlation coefficient of the spike trains for each population's neurons simulated on SpiNNaker (boxplots on the left) and NEST (boxplots on the right). The correlation coefficient is computed over the stimulation period on binned spike times with a bin width of 5 ms. Deep cerebellar nucleus cell (DCNC) is not included in the **(A)** excited firing rates and **(D)** correlation coefficient plots as it does not produce any spikes during the stimulation period, which those plots cover. The **(B)** ISI and **(C)** CV cover the entire simulation.

Spiking activity of the large-scale model simulations on the two platforms is further validated by comparing distributions of firing rates, inter-spike intervals, coefficients of variation, and correlation coefficients of individual neurons (Figure 5). An ideal correlation coefficient would have a value of 1 for each neuron pair. The correlation coefficients show all cell types except SC and BC have a median over 0.8, suggesting SpiNNaker and NEST produce comparable spike trains, in agreement with the ISI and CV comparisons. The spike trains produced by the two simulations are unlikely to match precisely due to differences between the simulation platforms such as the choice of arithmetic precision and solver. The differences in sub-threshold membrane potential computation identified in section 4.1.2 are amplified here due to the interactions between different cell types. SpiNNaker generally produces higher firing rates compared to NEST due to a combination of arithmetic and algorithmic error, e.g., error arising from the choice of fixed-point arithmetic and from the solver implementation.

### 4.3. Maximum Spike Packets Received per Timestep

Computing neuron updates on SpiNNaker constitutes a fixed overhead in the simulation (Rhodes et al., 2019). However, the CPU time spent processing synaptic events depends on the number of spikes arriving at a core in a particular timestep. To meet real-time processing requirements, the worst case scenario for individual timesteps must be understood. The maximum number of packets received in a timestep per core and per cell type throughout the simulation is thus investigated.

This section examines the effect of varying the amount of stimulation by controlling two parameters: the firing rate of selected glomeruli (*f*_*peak*_), and the stimulation radius, which controls the number of selected glomeruli. When one of the parameters is varied, the other is fixed at the default value as defined in section 2.2 (by default, *f*_*peak*_=150 Hz and stimulation radius = 140 μm; see section 4.2).

**Figure 6:**
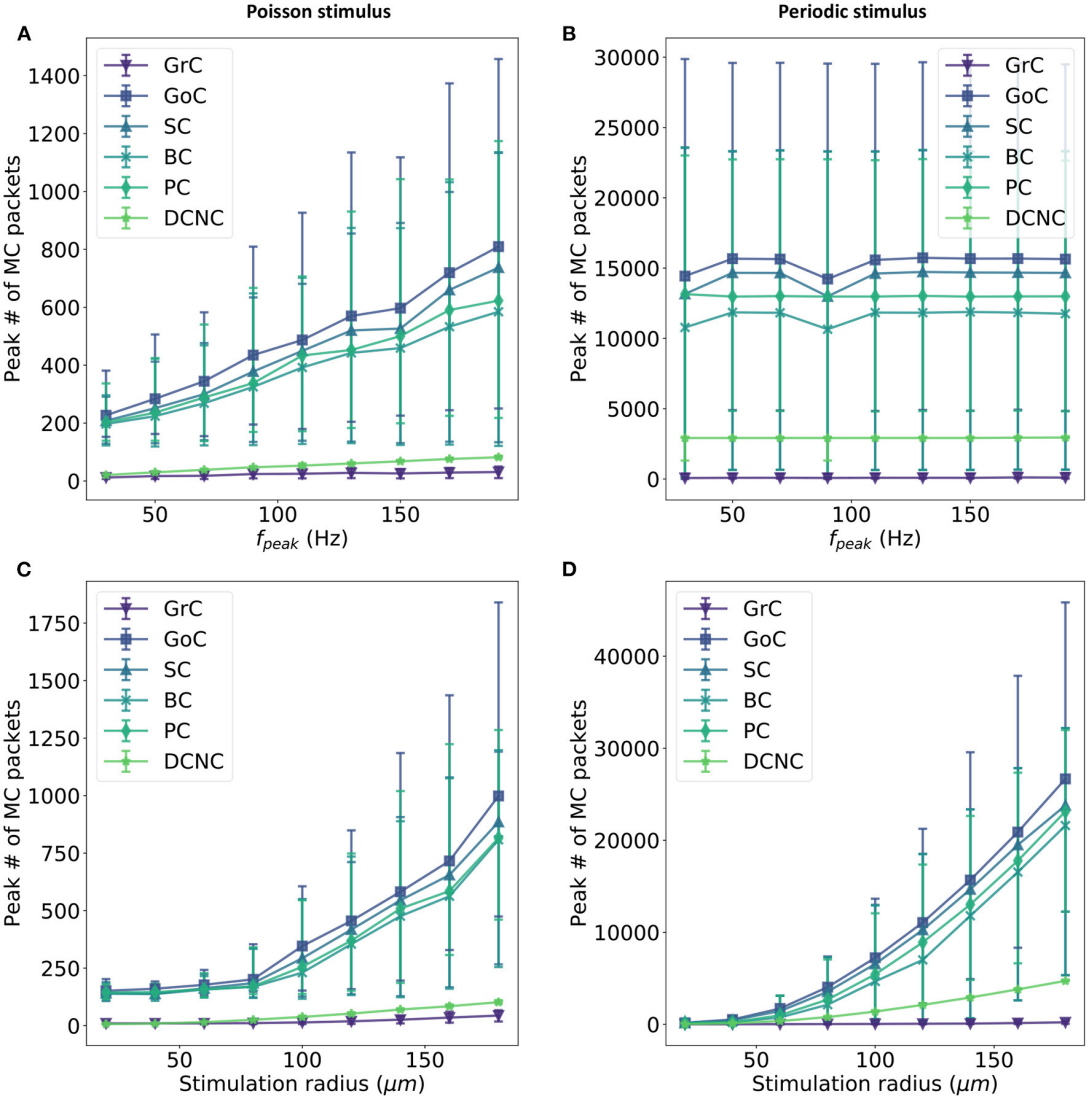
Relationship between input firing rate and maximum number of packets received by each core in a timestep. Input activity is controlled in two ways: a fixed set of Glomeruli is activated at a variety of firing rates (*f*_*peak*_)—**(A,B)**—or a variable set of glomeruli (controlled by the size of the stimulus selection radius) is activated at a fixed rate **(C,D)**. Two input stimulus encodings are used: Poisson input (left) and periodic input (right). The first row compares the maximum number of spikes received in a timestep for all other population in the model when varying the input firing for a fixed subset of Gloms, while the second row compares the same metric when the a fixed firing rate is maintained for a variable subset of Gloms.

The first parameter to be varied is *f*_*peak*_, moving from 30 to 200 Hz in increments of 20 Hz. Figures 6A,B reveal an approximately linear relationship between *f*_*peak*_ and the maximum number of multicast packets received by cores in a timestep. No change is seen in this peak activity when periodically stimulating the model, due to the synchronized activity of all stimulated cells (see section 3.1). This is expected and demonstrates that the resultant peak number of spikes is a direct consequence of the encoding of the stimulus and not an artifact of the model construction such as resonance or constructive interference.

The second parameter to be varied is the selection volume for Gloms while maintaining an input firing rate of 150 Hz. The stimulus radius is varied from 20 to 180 μ*m* in increments of 20 μm. This sweep has the effect of experimenting with as few as 14 selected glomeruli (0.2% of total Gloms) and as many as 4,718 (66.7% of total Gloms). When the input is encoded using periodic spikes, varying the stimulation radius increases the peak number of packets received by cores (c.f. Figures 6B,D) with the 95th percentile for GoC seeing over 40,000 packets in a single timestep. Under Poisson stimulation, GoC is still the most affected, with the 95th percentile now peaking at around 1,750 packets in a single timestep (Figure 6C).

There is a clear correlation between populations receiving afferent projections from GrC and very high numbers of packets received per timestep. DCNC and GrC achieve similar peaks when stimulated using Poisson-distributed spike trains (an average peak of 36 packets is recorded for GrC and 70 recorded for DCNC), but the difference increases when stimulated using periodic input, with GrC trailing DCNC by a significant margin (758 c.f. 2918 packets).

There is an increase of approximately 30× in the peak packet count received in a timestep for this model when going from Poisson to periodic stimulus for most populations: GoC, SC, BC, and PC; DCNC see a 40× increase, while the least affected are the GrC at 12×. Simultaneously, the peak conductance that PCs receive in a single timestep jumps to ~20 μS c.f. 1.7 μS in default conditions with Poisson stimulation. If the smallest weight a PC neuron would have to process is 2e-5 μS (the conductance transferred to the neuron by a single spike arriving on a pf-PC connection) and this conductance would be represented as a single bit, therefore to represent the peak conductance under periodic stimulation 19.93 bits of precision would be required.

The number of packets received per core is significantly affected by how neurons are organized on cores. Organizing neurons on cores according to their spatial location reduces the peak number of packets to be processed by each core by 41% compared to a random placement (averaging over all populations in the model). This result is consistent with previous modeling efforts that organized neurons in tiles according to neuron position and connectivity (Igarashi et al., 2019). Further details of the structure and positions of cells and their connections in 3D space, and the impact of this organization on the simulations performed on SpiNNaker are available in Supplementary Material (section 3).

Rhodes et al. (2019) saw empirically that using a paradigm where neuron and synapse processing is segregated onto different cores would allow the synapse cores to process ~20 packets in a timestep to maintain the real time requirement. Assuming that a similar technique could be employed here, populations such as GoC would require 50 synapse cores for each neuron core (currently containing 64 neurons) to process the 95th percentile peak number of packets, while PC would require 40 synapse cores attached to each neuron core (currently containing 1 neuron). Since SpiNNaker has a maximum of 18 cores per chip, this scaling approach will not be sufficient for GoC and PC. An extension will have to be implemented for this paradigm to function over multiple chips to allow for the activity levels seen in the cerebellum model.

Simply looking at the number of packets arriving at each core might not be sufficient to design an efficient software organization for cerebellar circuits. To extract optimal performance out of the on-chip DMA engines, cores which spend most of their time processing spiking activity will benefit from being placed on different chips from each other so as to prevent DMA contention (Rhodes et al., 2018). The following section highlights the current placement of populations and their peak DMA use, as well as the effect of placement on routing and communication.

### 4.4. Mapping the Cerebellum Onto SpiNNaker

All models have to be mapped onto SpiNNaker for simulation. Here, “mapping” is short-hand for the processes of partitioning, placing, and routing where populations are split into core-manageable sub-populations, which are then loaded to specific machine locations (section 2.1). These sub-populations communicate with each other as defined by the connectivity of the model, thus routers are set up to facilitate this communication. Designing a mapping from a 3D reconstruction of the cerebellum to the 2D surface of SpiNNaker is a challenge. Four approaches to perform this mapping are explored in Supplementary Material (section 3). The approach taking into account the position of cells in the volume both decreases the peak number of packets to be processed by individual cores while simultaneously increasing the efficiency of DMA transfers.

**Figure 7:**
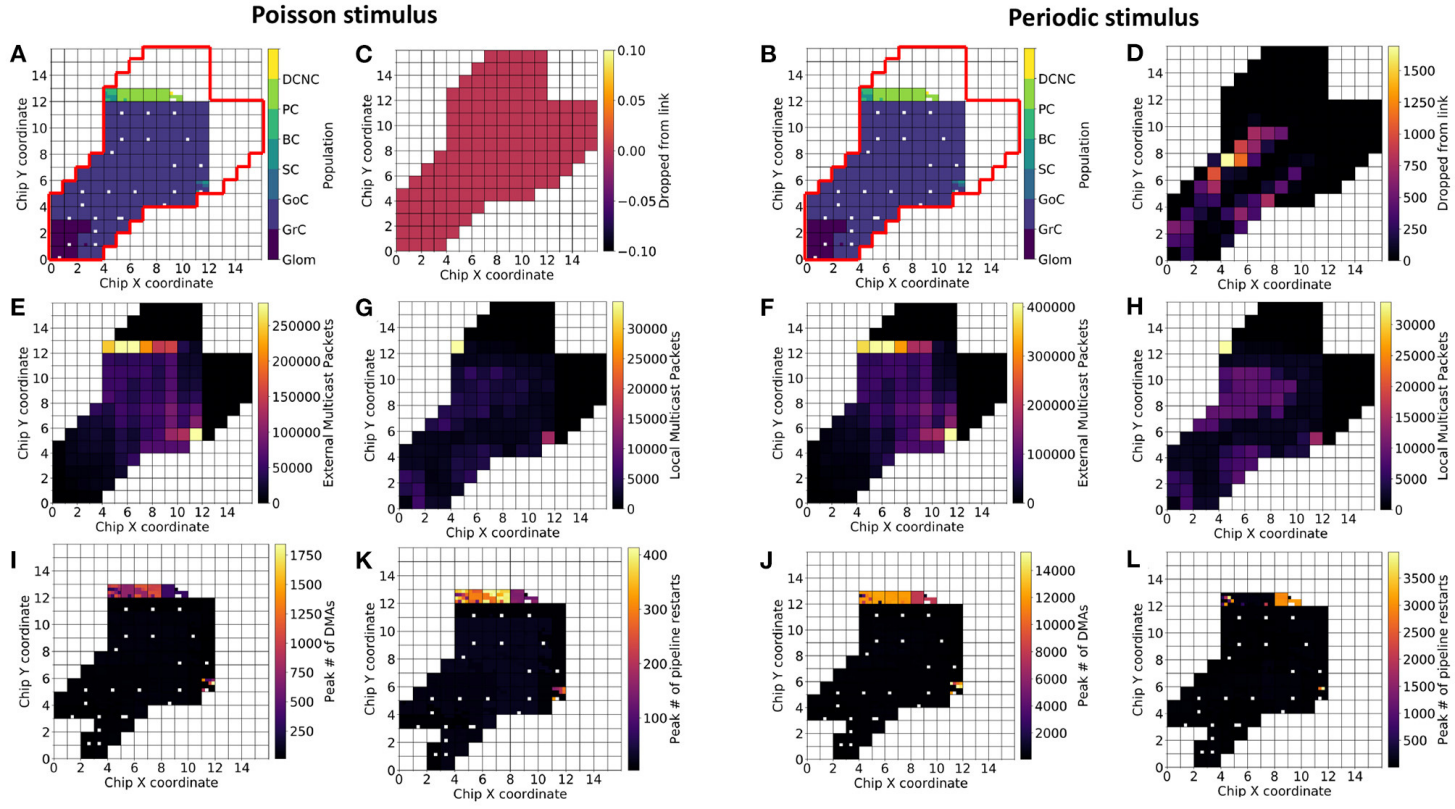
Population placement and statistics on three-board machines. The Cerebellum model requires ~1,583 cores for the current number of neurons per core (101 chips spread over three boards). For identical partitioning and placements with Poisson **(A)** and periodic **(B)** stimuli (red outline delimits the full extent of chips on the three-board system), the figure shows number of packets the routers dropped **(C,D)**, total number of external packets **(E,F)**, total number of local packets **(G,H)**, the maximum number of direct memory accesses (DMAs) in a timestep **(I,J)**, and the maximum number of processing pipeline restarts **(K,L)**. White squares within the 3-machine system used here correspond either to cores not active during the current simulation or cores that are permanently deactivated due to manufacture defects.

This section investigates (1) how neuronal populations are represented on the SpiNNaker machine and (2) how the spike traffic flows within the communication network. Figure 7 is divided into two halves: with the left, showing results for experiments driven with Poisson-distributed spikes, and the right, containing results for experiments driven with periodic stimuli (see section 3.1, for stimulus description).

Results from simulating the cerebellum model with identical placements are presented with Poisson (Figure 7A) and periodic (Figure 7B) stimulus. SpiNNaker routers are capable of processing all of the packets produced by the model driven with Poisson-distributed spikes, but dropped over 1,500 packets in some cases due to the synchronized arrival of packets (Figures 7C,D)—all dropped packets were subsequently reinjected into the network, however with no guarantee they will arrive within the same timestep. Neuronal populations simulated on chips whose routers drop packets are Glom and GrC.

SpiNNaker Routers process packets differently based on their source. Packets produced by a core on the current chip are defined as “local” from the perspective of that chip's router, while packets produced by a core on a different chip to the router are defined as “external,” regardless of the destination of the packet. Thus, it is possible to identify cores that produce many spikes, as well as routers which have to handle large numbers of packets produced elsewhere. The Poisson-driven model has a smaller peak of external packets (Figures 7E,F), however the relative ratio is similar between the two experimental scenarios. The increase in the upper bound of total external packets can be explained by (1) the increased activity of GrC producing more spikes—the increase in spikes produced is visible when looking at the total local packet count (Figures 7G,H)—and (2) the reinjected packets are counted once again as their respective type. Taking Figures 7A,E,G together reinforces the view that populations downstream of GrC, namely GoC, SC, BC, PC, are the main consumers of packets in the network and will require independent consideration to maximize model execution speed.

In addition to router-related statistics and peak numbers of packets received in a timestep, it is important to identify where and how information is transferred on each chip. Synaptic information is stored in chip-local, large and slow memory (SDRAM), and is transferred into core-local, small, and fast memory (DTCM) for processing. This transfer is performed through DMAs to enable the processor to continue working while data are transferred. The peak number of DMAs in a timestep in each scenario is presented in Figures 7I,J, and is consistent with the cores receiving the most packets in a timestep (not shown in the figure). The most affected cores here are those responsible for simulating SC, BC, and PC. This can lead to contention at a chips SDRAM controller, if chips have many cores issuing DMAs simultaneously (Rhodes et al., 2018). A future implementation may therefore benefit from preventing co-placement of neural populations that will issue large numbers of DMAs on the same chip.

The amortized cost of processing a batch of synaptic events is lower than processing them individually (Rhodes et al., 2018). It is therefore desirable to minimize the number of individual DMAs, and increase the amount of synaptic data transferred, thus favoring batch processing (Figures 7K,L). To this end, spike processing should be modified to prioritize neuron processing, followed by a period of synaptic processing, as opposed to the interleaved approach employed here enforcing a form of interrupt coalescing. Further optimization will seek to increase the length of synaptic rows, as this has been shown to increase the efficiency of individual DMAs. Practically, this would be achieved by increasing the number of neurons per core and ensuring neurons on a core share as many common afferents as possible.

## 5. Discussion

This work demonstrates the feasibility for large-scale, biologically plausible cerebellum simulations on neuromorphic hardware. The 3 components presented in section 2.1—programmable processing cores, routers exchanging information using multicast packets, and large shared memory—are the elements that make SpiNNaker neuromorphic platform ideally suited for simulating the cerebellum model presented in section 2.2. The cerebellum's large number of afferent synapses per neuron can be handled by the SDRAM present on every chip, a capability shared with few other neuromorphic platforms; its activity can efficiently be routed to the many target cells required to receive it; and the effect of the model's spiking activity on individual neurons can be modeled using efficient fixed point arithmetic on the programmable processing cores.

Numerical modeling was performed using fixed-point arithmetic representative of typical neuromorphic systems. However, to ensure accuracy when integrating neuronal dynamics, a sub-cycling technique was employed, together with synaptic weight normalization to avoid quantization errors. Additionally, it was found that representing model weights required at least 16 bits of precision when using fixed point arithmetic to produce comparable results to a baseline executed on the NEST simulator. Single cell experiments showed that SpiNNaker matches NEST in terms of sub-threshold voltage, precise spike times of neurons, and firing rates. Simulating experiments involving single cells with single and model-representative spiking input offered insights into modeling accuracy for individual cell types, which would be difficult to analyze in the large-scale model. The large-scale model was then translated from pyNEST into PyNN and simulated using sPyNNaker and NEST. Simulations show close agreement in terms of average firings rates before, during, and after stimulation, demonstrating that neuromorphic hardware is capable of simulating cerebellar neuronal dynamics and a neural circuit incorporating fan-ins of 28,000 synapses per Purkinje cell.

Future implementations of a large-scale cerebellum model may use more detailed models of point or compartimentalized neurons for increased biological plausibility (Masoli and D'Angelo, 2017; Teeter et al., 2018; Geminiani et al., 2019). Special consideration should be taken for such models, particularly with regard to the accuracy and speed of solving of more complex equations (Hopkins and Furber, 2015). While existing neuromorphic platforms may be able to cope with added complexity, they have been designed to accelerate mainly simplified neurons and synapses, and thus may not be the optimal platforms for more complex simulations. The next generations of neuromorphic devices, such as SpiNNaker2 (Mayr et al., 2019; Clark et al., 2020), may be better suited for the simulation of complex neuron models through the presence of floating point and transcendental function hardware acceleration.

Following model validation, the effect of stimulus encoding and magnitude was explored to extract requirements for future performance optimizations of model execution on SpiNNaker. This exploration revealed that the appropriate mapping of cells onto processing cores according to their spatial organization could reduce the peak number of packets received per core by 41% when averaged over all the populations in the model. Such reductions in peak communication is noteworthy as the combination of the large number of granule cells and realistic numbers of afferents per cell resulted in communication peaks 20–50× larger than previously established empirical real-time computational ability of SpiNNaker. Thus, analyzing the number of packets received by each processing core revealed the need for a different simulation paradigm with many processing cores spanning multiple chips dedicated solely to processing spiking activity. The time taken to simulate the model scales with its activity, rather than its size.

Future work will target the design and implementation of a software solution to allow simulation of the cerebellar circuit in real time based on the requirements gathered here. The ability to perform real-time cerebellum simulations will benefit both the neuroscience and robotics communities. Extended duration simulations will offer insights into the long-term operation of both the cerebellum and other neural circuits, in the future enabling simulation-based exploration of the neurophysiology of individual brain regions. The ability to co-simulate additional brains regions in real time, such as the cortex (Rhodes et al., 2019), will also enable research into the interaction between brain regions, e.g., the cortico-cerebellar loop. Furthermore, real-time neural simulation able to learn in an online fashion opens the door to exploration of brain-inspired circuits when embodied in robots, both as functional robotic controllers, and also as a path to study pathologies related to motor control.

## Data Availability Statement

The datasets presented in this study can be found in online repositories. The names of the repository/repositories and accession number(s) can be found at: Supporting data for a study toward a bio-inspired real-time neuromorphic cerebellum, EBRAINS, doi: 10.25493%2FPBYC-R3B.

## Author Contributions

BM assisted in translating the cerebellum model from pyNEST into PyNN. CC, SC, MH, and OR assisted in debugging, analyzing, and understanding the behavior of the model. AR debugged and validated the C executables running on SpiNNaker and implemented the sub-cycling behavior, while OR implemented the weight normalization technique used in this work. PB led the research and wrote the manuscript. FL, ED'A, and OR supervised this work. All authors reviewed and refined the final manuscript.

## Conflict of Interest

The authors declare that the research was conducted in the absence of any commercial or financial relationships that could be construed as a potential conflict of interest.

## Footnotes

^1^“Supporting data for a study towards a bio-inspired real-time neuromorphic cerebellum”. Available online at: https://search.kg.ebrains.eu/instances/Dataset/069e4718-2b4a-4056-9c8f-e646a841b2dd

^2^https://elephant.readthedocs.io
