# Supplementary material for: Towards a Bio-Inspired Real-Time Neuromorphic Cerebellum
Source: Front Cell Neurosci. 2021 May 31;15:622870. doi: 10.3389/fncel.2021.622870 (PMC8202688; doi:10.3389/fncel.2021.622870)
Supplement: Supplementary file 1 [file Data_Sheet_1.PDF]

frontiers

Supplementary Material

1 NEURON AND SYNAPSE DYNAMICS

Neurons throughout this work are simulated as conductance-based leaky integrate and fire (LIF) units. The sub-threshold dynamics of individual neurons are dictated by Equations S1 and S2. The SpiNNaker neural simulation software 'sPyNNaker' uses the same equation for both current and conductance-based inputs. This is covered in detail by Rhodes et al. (2018), but is also presented here for clarity. Equations S1 and S2 are reproduced from Rhodes et al. (2018) for current-based LIF cells:

$$\frac{dV}{dt} = -\frac{V - (E_l + R_{mem}I(t))}{\tau_m} \tag{S1}$$

$$\frac{dI_{syn}}{dt} = -\frac{I_{syn}}{\tau_{syn}} + \delta(t - t^j) \tag{S2}$$

where V is the membrane potential, I is the (total) input *current* (this includes the synaptic current contribution I_{syn}, and direct current I_{DC}), R_{mem} is the membrane resistance, τ_m is the membrane leak time constant and E_l is the membrane leak potential. The input current at time t is computed by converting the input conductance for each synapse type (excitatory and inhibitory) through Equation S3:

$$I(t) = G(t)(E_{rev} - V) \tag{S3}$$

where $G(t)$ is the input conductance at time t, E_{rev} is the reversal potential for the chosen synapse type and V is the membrane potential. Equations S1 & S2 are solved analytically, with Equation S1 assuming the input current across a timestep is constant (Dayan and Abbott, 2002). This yields the sequential updates detailed in Equation S4, enabling advancement of all state variables from time t to $t + \Delta t$.

$$\begin{aligned} G_{t+\Delta t} &= G_t \mathrm{e}^{-\frac{\Delta t}{N_{sc}\tau_{syn}}} + \sum_j w_{ij}^n \delta(t - t^j) \\ I_{t+\Delta t}^n &= G_{t+\Delta t}(E_{rev} - V_t) + I_{DC}^n \\ V_{t+\Delta t} &= (E_l + I_{t+\Delta t}^n) - \mathrm{e}^{-\frac{\Delta t}{N_{sc}\tau_m}}((E_l + I_{t+\Delta t}^n) - V_t) \end{aligned} \tag{S4}$$

Due to the coupling between I and V in conductance-based neurons, when the membrane potential of a neuron changes rapidly this sequential update scheme can become unstable. An additional term N_{sc} is therefore included in Equation S4, which details the number of sub-cycles within a state update. This effectively allows a smaller simulation timestep to be used within a state update, helping ensure stability at the expense of additional computation. In addition to this sub-cycling scheme, normalisation of the synaptic and direct input current prior to simulation is performed, by dividing through by the membrane conductance ($\frac{C_m}{\tau_m}$). Terms employing this normalisation are denoted by the superscript n in Equation S4. This scaling helps increase the precision of synaptic decay operations (i.e. solving Equation S2) which typically involve small quantities, by scaling up these values to avoid quantisation within the fixed-point arithmetic.

2 USING 16 BIT WEIGHTS

Some design decision were taken for SpiNNaker to bring down cost and power for each chip; it uses 32-bit ARM processors lacking a hardware floating point unit. Software floating point libraries are available, however they are generally avoided for two reasons: they occupy large amounts of instruction tightly-coupled memory (ITCM, 32 kB) and are expensive in terms of clock cycles. Optimised libraries employing fixed point arithmetic types have been developed over the years to forgo the need for floating point operations, however this is not without caveats. Weight values are stored as 16-bit integers that are appropriately scaled so as to be able to correctly add different weights from different connections together to compute the total synaptic contribution for individual neurons in any one time step. This integration is performed in a 64 element circular buffer structure (as many elements as delay time steps in the network, i.e. this network can handle a delay of up to 6.4 ms at 0.1 ms time steps). Identical, but separate structures are maintained for excitatory and inhibitory connections, with individual buffer entries represented as 16-bit integers, to help preserve memory. These data structures are stored in core-local, relatively fast, but relatively small data tightly-coupled memory (DTCM, 64 kB) rather than relatively slow and large chip-local SDRAM (128 MB).

At each time step, the accumulated value contained within the subsequent element of the circular buffer is cast to a 32 bit value in the S16.15 format (ISO/IEC, 2008) for use in updating the sub-threshold potential of the neuron. The conversion is done by left shifting the value from the circular buffer by a pre-computed shift amount. The appropriate shift value is selected for the fixed point value to be represented. In other words, the shift value indicates the position in the number which separates the integer and fractional parts. Correctly computing the shift values requires understanding of the range the number is required to span. In this context, a shift of 0 describes numbers which have 1 integer bit, while a shift of 15 describes natural numbers without a fractional part (Rhodes et al., 2018).

An estimate of the peak activity is generally used to predict the maximum weight which individual circular buffer elements have to represent. In the current work, we can extract the peak activity per time step from the baseline simulation in NEST, and feed those values into our computation of an appropriate shift value. Table S1 reveals the empirical maximum number of spikes arriving at individual neurons in any one time step. The values are computed independently for each projection, but they are used as if they were occurring during the same time step. The excitatory and inhibitory shifts are computed according to Eq. S5:

$$\begin{aligned} \text{shift} &= \text{ceil}(\log_2(w)) \\ \text{scale} &= 2^{16-(\text{shift}+1)} \end{aligned} \tag{S5}$$

where w is the estimated maximum weight (conductance) to be represented, shift encodes the position of the decimal separator, while scale is the value which when multiplied with an entry of the circular buffer results in the synaptic contribution received by a neuron at the current time step. The full pipeline is detailed in full by Rhodes et al. (2018).

To obtain the spike counts in Table S1, a baseline NEST simulation was presented with a particular stimulus file. Based on the activity recorded in that simulation, the maximum number of spikes in a time step could be identified for each neuron and from each projection. All weights in a projection are equal in this experiment, so the maximum weight contributed by each projection is simply the number of spikes multiplied by the weight of a connection. The same weight scaling is applied to all weights arriving at a post-synaptic core simulating a slice of a population. Thus, the total synaptic contribution per type is

Post-synaptic population	Connection name	Maximum number of spikes in a time step	Max. excitatory weight (μS)	Max. inhibitory weight (μS)	Excitatory shift	Inhibitory shift
**GrC**	Glom – GrC	5	0.045000			
	GoC – GrC	4		-0.020000		
	**Total**		**0.045000**	**-0.020000**	**5**	**4**
**GoC**	Glom – GoC	8	0.016000			
	aa – GoC	28	0.560000			
	pf – GoC	51	0.020400			
	GoC – GoC	25		-0.200000		
	**Total**		**0.596400**	**-0.200000**	**8**	**6**
**SC**	pf – SC	53	0.010600			
	SC – SC	4		-0.008000		
	**Total**		**0.010600**	**-0.008000**	**4**	**3**
**BC**	pf – BC	51	0.010200			
	BC – BC	5		-0.012500		
	**Total**		**0.010200**	**-0.012500**	**4**	**4**
**PC**	aa – PC	23	1.725000			
	pf – PC	621	0.012420			
	SC – PC	7		-0.059500		
	BC – PC	6		-0.054000		
	**Total**		**1.737420**	**-0.113500**	**7**	**5**
**DCNC**	Glom – DCNC	6	0.000036			
	PC – DCNC	6		-0.000180		
	**Total**		**0.000036**	**-0.000180**	**0**	**0**

Table S1. Reference peak number of spikes and associate weights assuming peak activities from different projections arrive synchronously. The peak number of spikes for each projection was obtained for a particular stimulus after simulation on NEST. The same stimulus was then used in the SpiNNaker simulations.

computed and that is the weight w used in Eq. S5 to compute the required shift and scaling for all weights terminating at the same post-synaptic population.

Deciding on scaling factors for weights based on maximum expected synaptic input in a time step attempts to avoid saturating circular buffer entries. In other words, the choice of scaling aims to avoid losing information when processing peak activity and the associated peak conductance. However, focusing purely on the peak activity can mean that relatively small weights get rounded down to 0. This effect, if not accounted for, is encountered here for pf-PC connections. Thus, the excitatory shifts presented in Table S1 are lower by 1. In practice our assumption that all connections are active at the same time is conservative, meaning this relaxation is valid for the simulated model.

Table S2 shows that 16 bits of precision is sufficient to represent most of the weights in the cerebellar network within 5% of the prescribed value. The outlier is the weight associated with pf-PC connections that is 37.61% larger than the prescribed weight. Thus, the weight for pf-PC connections is represented by setting high the least significant bit in our 16 bit representation. In order to accurately represent the weight of pf-PC given the peak conductance, a minimum of 16.41 bits of resolution would be required. We thus accept the potential for this buffer to be saturated.

Connection name	Prescribed weight (μS)	Weight on SpiNNaker μS (Error %)
Glom – GrC	9.0000e-3	9.0000e-3 (0.00%)
Glom – GoC	2.0000e-3	2.0074e-3 (0.37%)
Glom – DCNC	6.0000e-6	6.0039e-6 (0.07%)
aa – GoC	2.0000e-2	1.9990e-2 (–0.05%)
pf – GoC	4.0000e-4	3.9583e-4 (1.04%)
pf – SC	2.0000e-4	2.0020e-4 (0.10%)
pf – BC	2.0000e-4	2.0020e-4 (0.10%)
aa – PC	7.5000e-2	7.4996e-2 (0.01%)
pf – PC	2.0000e-5	2.7521e-5 (37.61%)
GoC – GrC	–5.0000e-3	–5.0002e-3 (0.00%)
GoC – GoC	–8.0000e-3	–8.0015e-3 (0.02%)
SC – SC	–2.0000e-3	–2.0000e-3 (0.00%)
SC – PC	–8.5000e-3	–8.4972e-3 (–0.03%)
BC – BC	–2.5000e-3	–2.5000e-3 (0.00%)
BC – PC	–9.0000e-3	–8.9995e-3 (–0.01%)
PC – DCNC	–3.0000e-5	–3.0020e-5 (0.07%)

Table S2. Weight representation on SpiNNaker. The prescribed weights are transformed from a 64-bit double-precision floating point representation to a 16-bit fixed point representation, which is then read from the machine and cast back to double precision for comparison purposes (“Weight on SpiNNaker” column). Connections are ordered first by their afferent, pre-synaptic population, then by their efferent, post-synaptic population. The error is computed by taking the difference between the absolute value of the weight on SpiNNaker, subtracting the presribed weight and computeing the percentage of that difference with respect to the presribed weight.

3 NETWORK STRUCTURE AND ROUTING

The scaffold model described by Casali et al. (2019) produced the network used in the experiments in this work. The spatial positions of all the cells in the network are presented in Fig. S1. The problem facing many-core systems is how to partition and map these neurons onto hardware resources so as to maximise utility. In the case of SpiNNaker, the task is to map neurons in each population onto a 2D sheet of processing cores while wanting to minimise the number of stray packets per core and maximise the utilisation of the DMA engines, among other possible considerations. Stray packets are defined as multicast packets which when routed to a particular post-synaptic core do not affect any of the neurons on that core. This occurs on SpiNNaker because the multicast routes link cores together and not individual neurons. In other words, if two cores are connected by a route, all neurons on the source core packets will be sent to the target core, which then has to appropriately process the spikes which target no neurons.

To better understand the impact of network structure on communication, the relation between neuron identifier (a population-local unique number) and the cell position in the defined volume is explored. For brevity, reporting is limited to findings for granule cells (GrCs), but these generalise well to other cell types. Initially, GrCs are given an auto-incrementing ID as they are placed in the volume beginning at low Z coordinate and covering the XY plane. The numbering of atoms is shown in Fig. S2 where dark colouring corresponds to the lower end of neuron IDs and brighter colours corresponds to the higher end of neuron IDs. This initial mapping is compared with 3 others: random, Hilbert curve, and grid. The random mapping reassigns a random ID for each neuron in the space with no relation to its position. The Hilbert space filling curve approach relies on dividing the volume into voxels as defined by a Hilbert curve and traversing them in order to number the neurons. Finally, the grid-based approach projects the coordinates of neurons in the

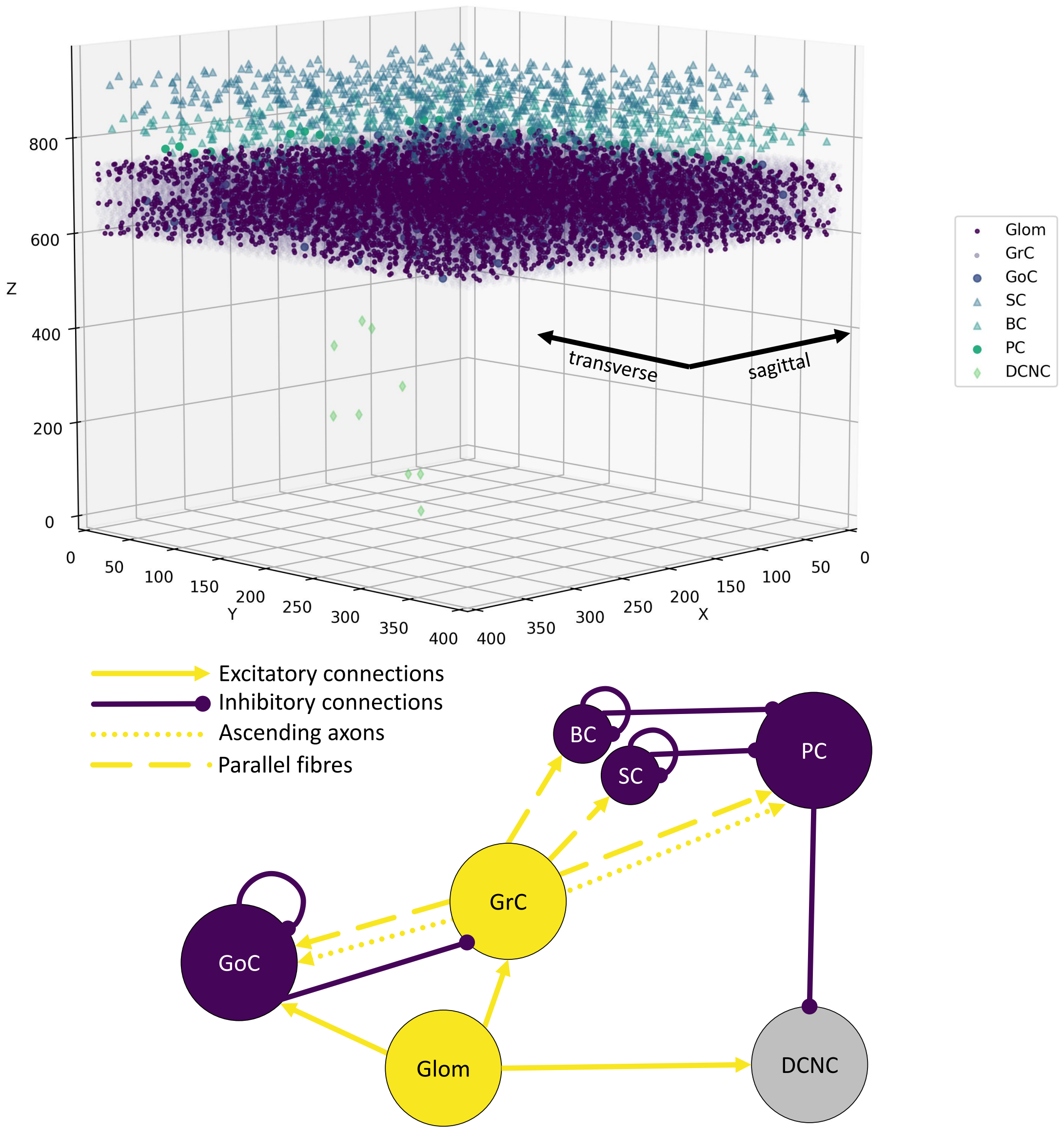

Figure S1: Cell positions within volume and logical network architecture.

XY plane and dividing the space into a number of rows (23) and columns (9). Cells are then numbered by first iterating over columns then rows.

The latter mapping was inspired by the columnar organisation of afferents to PCs. Parallel fibre connections from GrC to PC can be seen as roughly dividing the volume into 3 columns, with a similar finding for ascending axons. Figure S3 shows 1 of the 3 columns of PCs with their respective afferents for pf-PC (A) and aa-PC (B). The columns defined by the PC and their afferents contain approximately 32k GrCs, with over 75% of these cells sending efferents to all of the PCs in that column and only 4k GrCs being represented in two adjacent columns.

The other parallel fibre connections display qualitatively similar behaviour in that the afferent granule cells form columns for each post-synaptic target they connect with. They differ in scale – pf-PC connections

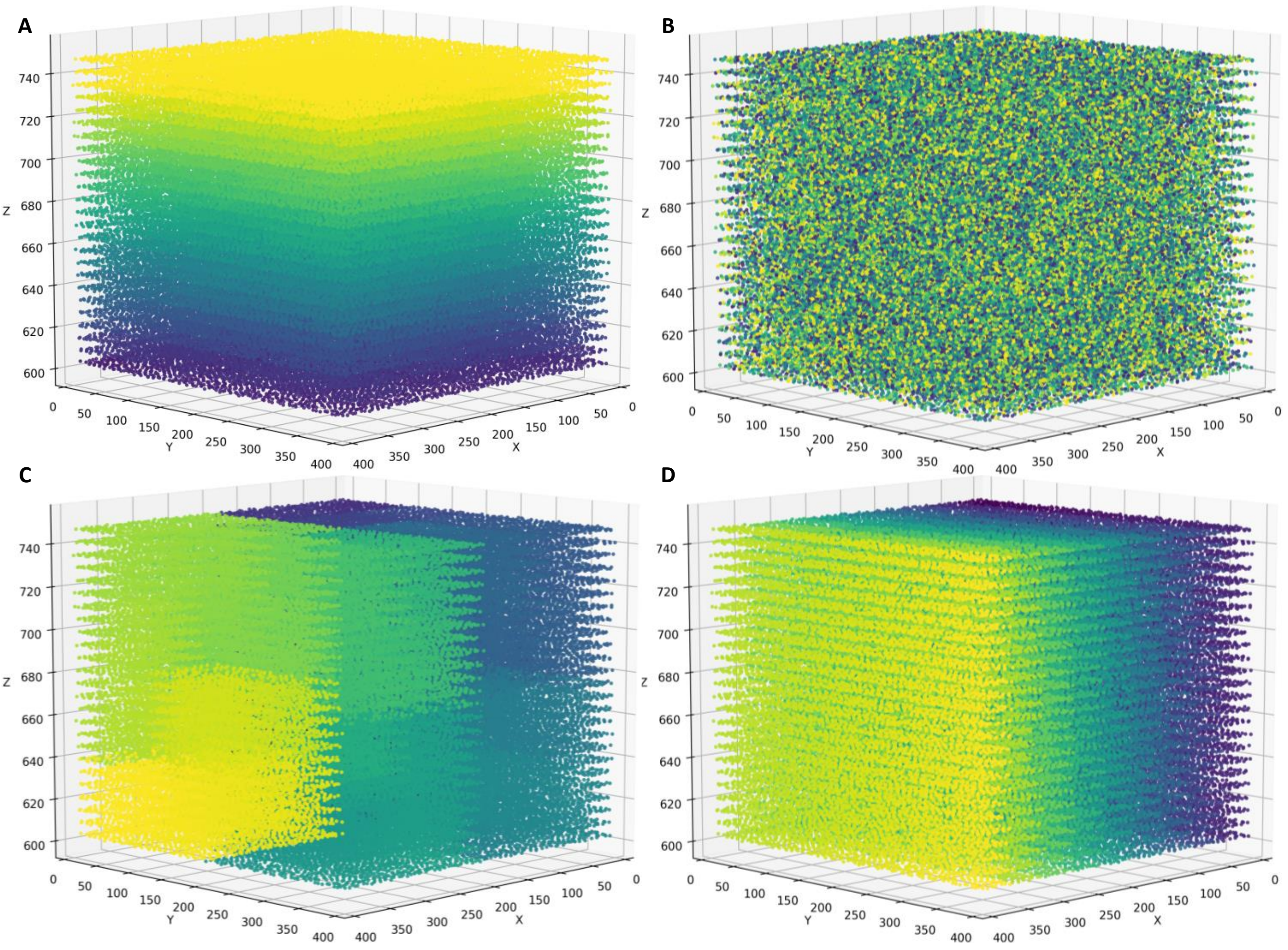

Figure S2: Granule cell rasterisation methods. A. Initial mapping. B. Random mapping. C. Hilbert space filling curve. D. Grid-based mapping.

are the most numerous in the network – and have the largest spread along the X axis – pf-PC connection afferents divide the space into thirds, while the other parallel fibre connections are narrower.

The large-scale network was simulated with the 4 proposed mechanisms to associate IDs with neurons. Each simulation setup is run 5 times with different stimuli. The number of packets received by each processing core is recorded, along with the range of post-synaptic neurons that packet targeted. The ranges used are a: no post-synaptic neurons, so these are stray packets; b: 1 post-synaptic neuron targeted; c: 2-5 post-synaptic neurons targeted; d: over 5 post-synaptic neurons targeted. The results are summarised in Table S3.

Ideally, cores should not receive stray packets as these incur a 5 μs overhead to process without providing any information to the core. Further, a more optimised mapping requires fewer total packets flying through the network – fewer packets could be used to transmit the same number of spikes to appropriate post-synaptic cores if the packet targets many or all of the neurons on individual post-synaptic cores. In short, the goal of the mapping should be to minimise the number of stray (a) and total packets, while maximising

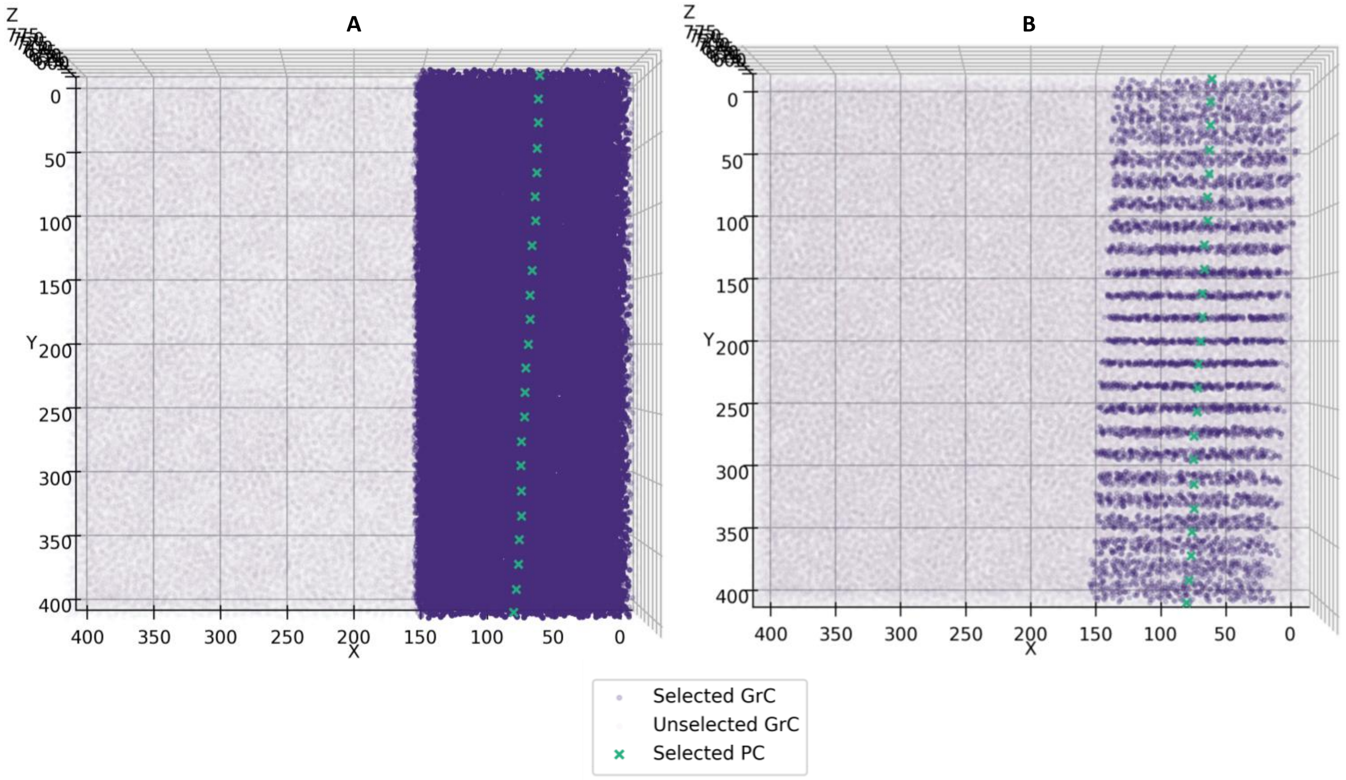

Figure S3: Afferents for a third of PCs from A. pf-PC and B. aa-PC viewed top-down in the $400 \times 400 \times 900\mu$m volume. The selected PCs receive connections from the selected GrCs, but not from the unselected ones.

the number of post-synaptic targets (b, c, d). The latter is because the DMA engines built into SpiNNaker are designed for bulk transfer of data from memory (for full details see Rhodes et al. (2018)).

On average, grid mapping outperforms all other mapping approaches explored here for total number of packets received. Grid mapping also introduces a reduction in the number of stray packets in the network, and increases the number of packets which target more than 5 post-synaptic neurons on a core (case d). However, grid mapping does not offer improvements everywhere. DCNC particularly sees little difference when using any of the proposed mapping approaches. This can be attributed both to the small number of neurons, the fact that the population occupies a single processing core and the population's relatively small synaptic fan-in.

When compared to random mapping, grid mapping reduces the peak number of packets received by cores by 41% on average for all populations. DCNC sees the least improvement using grid mapping, decreasing the number of packets by 3.8%. The biggest improvement is seen by GrC (67.13%), with good improvements experienced by all other populations: GoC (37.21%), SC (41.83%), BC (47.35%) and PC (50.39%). We conclude that many-core systems using multicast and core-level routing, such as SpiNNaker, would benefit from mapping cerebellum neuron IDs according to their spatial location.

REFERENCES

Casali, S., Marenzi, E., Medini, C., Casellato, C., and D'Angelo, E. (2019). Reconstruction and simulation of a scaffold model of the cerebellar network. *Frontiers in Neuroinformatics* 13, 1–19. doi:10.3389/fninf.2019.00037

Population	Avg. metric	Initial mapping	Random mapping	Hilbert mapping	Grid mapping
**GrC**	a	34.31±7.61	66.38±7.11	23.42±8.91	**21.87±7.96**
	b	**0.15±0.41**	1.96±1.45	0.06±0.24	**0.15±0.43**
	c	0.29±0.60	0.03±0.18	0.10±0.34	**0.36±0.76**
	d	**0.11±0.35**	0.00±0.00	**0.11±0.35**	0.09±0.32
	Total	34.86±7.60	68.37±6.88	23.68±9.02	**22.47±8.27**
**GoC**	a	1313.85±296.89	1118.90±262.10	740.40±90.68	**462.35±193.00**
	b	**682.45±128.57**	589.15±140.74	528.20±118.23	434.85±280.56
	c	314.05±132.39	267.00±118.38	314.75±97.92	**321.60±241.26**
	d	0.65±1.18	0.70±0.80	4.75±4.35	**21.75±19.78**
	Total	2311.00±227.18	1975.75±225.54	1588.10±235.41	**1240.55±702.56**
**SC**	a	687.38±109.22	565.82±75.11	461.14±134.02	**389.56±185.00**
	b	64.68±22.22	**186.34±41.96**	37.74±19.29	23.26±17.59
	c	129.90±59.01	**212.22±69.34**	129.46±77.56	76.76±53.06
	d	53.52±38.04	2.04±3.51	54.06±34.14	**72.54±60.36**
	Total	935.48±177.35	966.42±114.84	682.40±208.21	**562.12±281.66**
**BC**	a	673.00±96.36	594.42±106.47	471.48±94.52	**336.14±180.66**
	b	56.50±27.48	**179.98±34.85**	32.50±14.29	16.38±15.38
	c	152.64±40.75	**189.48±58.43**	98.16±37.73	77.52±62.20
	d	33.42±25.03	1.18±2.97	54.40±31.39	**78.02±61.68**
	Total	915.56±141.60	965.06±115.20	656.54±149.31	**508.06±298.07**
**PC**	a	445.20±97.87	801.09±208.87	183.01±119.67	**150.79±89.16**
	b	400.28±204.95	328.04±195.23	391.00±189.80	**409.38±237.55**
	c	0.00±0.00	0.00±0.00	0.00±0.00	0.00±0.00
	d	0.00±0.00	0.00±0.00	0.00±0.00	0.00±0.00
	Total	845.48±278.08	1129.13±132.50	574.01±271.73	**560.17±277.15**
**DCNC**	a	**50.00±2.92**	54.40±4.67	52.40±2.88	52.40±4.16
	b	**14.00±3.08**	11.20±5.20	10.80±1.92	11.00±2.55
	c	**2.00±1.41**	1.60±0.49	1.80±1.10	1.20±0.84
	d	0.00±0.00	0.00±0.00	0.00±0.00	0.00±0.00
	Total	66.00±4.00	67.20±2.77	65.00±1.22	**64.60±3.91**

Table S3. Expected peak number of packets received and processed per core. All populations are simulated with at most 64 neurons per core, except PC which is simulated with 1 neuron per core. Highlighted entries in the table correspond to the best average values in that respective row. Small values are best for case a and total numbers, while large numbers are best for all other metrics (b, c and d). The ranges used are a: 0 post-synaptic neurons targeted; b: 1 post-synaptic neuron targeted; c: 2-5 post-synaptic neurons targeted; d: over 5 post-synaptic neurons targeted.

Dayan, P. and Abbott, L. (2002). Theoretical Neuroscience: Computational and Mathematical Modeling of Neural Systems (Computational Neuroscience). *Journal of Cognitive Neuroscience* , 480doi:10.1016/j.neuron.2008.10.019

Hopkins, M. W. and Furber, S. B. (2015). Accuracy and Efficiency in Fixed-Point Neural ODE Solvers. *Neural computation* 1872, 1840–1872. doi:10.1162/NECO

ISO/IEC (2008). *TR 18037:2008 Programming languages — C — Extensions to support embedded processors*. Tech. rep., ISO/IEC

Rhodes, O., Bogdan, P. A., Brenninkmeijer, C., Davidson, S., Fellows, D., Gait, A., et al. (2018). sPyNNaker : A Software Package for Running PyNN Simulations on SpiNNaker. *Frontiers in Neuroscience* 12. doi:10.3389/fnins.2018.00816
